# Deconwolf enables high-performance deconvolution of widefield fluorescence microscopy images

**DOI:** 10.1038/s41592-024-02294-7

**Published:** 2024-06-06

**Authors:** Erik Wernersson, Eleni Gelali, Gabriele Girelli, Su Wang, David Castillo, Christoffer Mattsson Langseth, Quentin Verron, Huy Q. Nguyen, Shyamtanu Chattoraj, Anna Martinez Casals, Hans Blom, Emma Lundberg, Mats Nilsson, Marc A. Marti-Renom, Chao-ting Wu, Nicola Crosetto, Magda Bienko

**Affiliations:** 1 Department of Microbiology, Tumor and Cell Biology, Karolinska Institutet, Stockholm, Sweden; 2 Science for Life Laboratory, Stockholm, Sweden; 3 CNAG-CRG, Centre for Genomic Regulation (CRG), Barcelona Institute of Science and Technology (BIST), Barcelona, Spain; 4 Department of Biochemistry and Biophysics, Stockholm University, Stockholm, Sweden; 5 Department of Genetics, Harvard Medical School, Boston, MA USA; 6 School of Engineering Sciences in Chemistry, Biotechnology and Health, KTH - Royal Institute of Technology, Stockholm, Sweden; 7 Department of Applied Physics, Royal Institute of Technology, Solna, Sweden; 8 Department of Bioengineering, Stanford University, Stanford, CA USA; 9 Department of Pathology, Stanford University, Stanford, CA USA; 10 Chan Zuckerberg Biohub San Francisco, San Francisco, CA USA; 11 Centre for Genomic Regulation (CRG), Barcelona Institute of Science and Technology (BIST), Barcelona, Spain; 12 Pompeu Fabra University, Barcelona, Spain; 13 ICREA, Barcelona, Spain; 14 Wyss Institute, Harvard Medical School, Boston, MA USA; 15 Human Technopole, Milan, Italy; 16 Present Address: Acuity Spatial Genomics, Newton, MA USA

**Keywords:** Wide-field fluorescence microscopy, Software

## Abstract

Microscopy-based spatially resolved omic methods are transforming the life sciences. However, these methods rely on high numerical aperture objectives and cannot resolve crowded molecular targets, limiting the amount of extractable biological information. To overcome these limitations, here we develop Deconwolf, an open-source, user-friendly software for high-performance deconvolution of widefield fluorescence microscopy images, which efficiently runs on laptop computers. Deconwolf enables accurate quantification of crowded diffraction limited fluorescence dots in DNA and RNA fluorescence in situ hybridization images and allows robust detection of individual transcripts in tissue sections imaged with ×20 air objectives. Deconvolution of in situ spatial transcriptomics images with Deconwolf increased the number of transcripts identified more than threefold, while the application of Deconwolf to images obtained by fluorescence in situ sequencing of barcoded Oligopaint probes drastically improved chromosome tracing. Deconwolf greatly facilitates the use of deconvolution in many bioimaging applications.

## Abstract (web summary)

Deconwolf is a computationally efficient and user-friendly software tool for fluorescence microscopy image deconvolution that improves the analysis of diverse fluorescence in situ hybridization methods and can handle large datasets.

## Main

In fluorescence microscopy, deconvolution is used to enhance image sharpness and contrast by reversing the optical distortions that occur as light travels through a microscope^1^ (Supplementary Note 1). Although several deconvolution tools are available, their widespread adoption has been hindered by high licensing costs (for commercial software) and the inability of existing tools to process large image datasets. Recent attempts to improve the Richardson–Lucy method^2,3^ use an unmatched back projector^4^, graphics processing units (GPUs)^5^ or pre-filtering of the input data^6^. Machine learning has also been applied to deconvolve fluorescence microscopy images^7^. However, these methods rely on proprietary software, vendor-specific hardware or tailored data training, and require high expertise to be implemented. Thus, there is a strong need for open-access, easy-to-operate and computationally efficient tools for fluorescence microscopy image deconvolution.

An important emerging application of deconvolution is in the booming field of spatial biology. Microscopy-based spatial omics, such as in situ spatial transcriptomics (ISST)^8^, and fluorescent in situ RNA sequencing (FISSEQ)^9^, potentially enable the visualization of hundreds or even thousands of different DNA loci and RNA species in their native tissue context. However, these methods have two major limitations: first, high numerical aperture (NA) objectives are required to localize the (near-)diffraction limited fluorescent signals that these techniques typically generate, significantly limiting the portion of a sample that can be effectively imaged; and second, when the target molecules are crowded, it becomes difficult to resolve them even using high-NA objectives (Supplementary Note 2). One approach to counteract this problem is the use of expansion microscopy^10^ with or without super-resolution microscopy^11^. However, these methods cannot be easily scaled up. Another approach is to use deconvolution, however, currently available deconvolution tools cannot overcome the inability to resolve crowded signals. Therefore, novel approaches are needed to maximize the amount of information that can be obtained from such techniques.

Towards this goal, we develop Deconwolf, an easy-to-operate and computationally efficient deconvolution software that can process any type of fluorescence microscopy signal. We show that Deconwolf outperforms the two most popular deconvolution tools, and showcase its technical performance on crowded images generated by single-molecule RNA fluorescence in situ hybridization (smFISH)^12^ and high-resolution DNA FISH (iFISH)^13^. By applying Deconwolf to images of tissue sections processed using smFISH, we show that individual transcripts can be accurately counted even when the images are acquired using low-magnification (×20) air objectives. Finally, we apply Deconwolf to ISST^8^ and fluorescence in situ sequencing of barcoded Oligopaint probes (OligoFISSEQ)^14^, demonstrating that Deconwolf increases the amount of information that can be obtained with these techniques. In summary, Deconwolf is a user-friendly tool that greatly improves the sensitivity and spatial resolution of imaging-based spatial omics and has numerous potential applications in bioimaging, enabling the processing of terabyte-scale data in realistic times.

## Results

### Deconwolf implementation and benchmarking

**Fig. 1:**
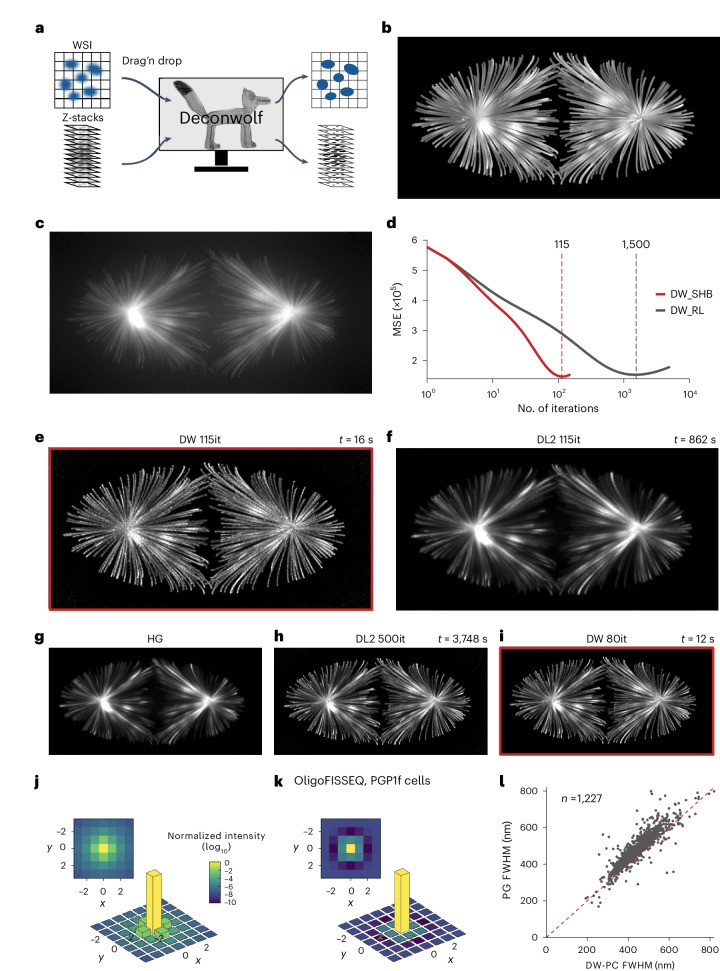
Implementation and benchmarking of Deconwolf. **a**, Schematic Deconwolf workflow. WSI, whole-slide image. **b**,**c**, In silico generated microtubule images before (ground truth) (**b**) and after adding artificial noise to simulate a real image (**c**). Maximum z-projection is shown. **d**, MSE after deconvolving the image in **c** using the default Deconwolf mode with scaled heavy ball^15^ acceleration (DW_SHB), or Deconwolf based on the classic Richardson–Lucy deconvolution method (DW_RL)^2,3^. The dashed vertical lines indicate the number of iterations needed to reach the minimum MSE. **e**, As in **c** after deconvolution with Deconwolf (DW) using default settings. it, number of iterations. *t*, deconvolution time measured on an 8-Core AMD Ryzen 7 3700X machine. **f**, As in **c** using DeconvolutionLab2 (DL2) with default settings at 115 iterations. **g**, As in **e** using Huygens Professional (v17.04) with default settings (HG). **h**, As in **f** at 500 iterations. **i**, As in **e** at 80 iterations. **j**,**k**, 2D profiles of the PSF used to generate the plot in **i**, calculated using either the PSF Calculator implemented in Deconwolf (DW-PC) (**j**) or PSF Generator (PG)^20^ (**k**). The bottom plots show 3D renderings of the corresponding 2D heatmap shown on the top. (**l**) FWHM of fluorescence dots in images previously generated by OligoFISSEQ^14^ deconvolved using the PSFs in **j** and **k**. Dashed red line, bisector of the angle between the plot axes. Each gray dot corresponds to one OligoFISSEQ dot. *n*, number of dots. Deconwolf-deconvolved images are framed in red. A link to the Source Data for this figure is provided in the Data Availability statement . Source Data

Deconwolf can be run on an ordinary laptop computer and features an intuitive interface through which multiple z-stacks or whole-slide images can be processed after specifying a few intuitive parameters (Fig. 1a). Deconwolf builds on the Richardson–Lucy method^2,3^ with three crucial improvements: first, highly efficient implementation of the scaled heavy ball (SHB)^15^ acceleration method to reduce the number of required Richardson–Lucy iterations; second, a high-precision point spread function (PSF) calculator; and third, automatic lateral and axial boundary handling with minimal artifacts (Methods). The entire Deconwolf package can be freely downloaded from https://deconwolf.fht.org/.

**Extended Data Fig. 1:**
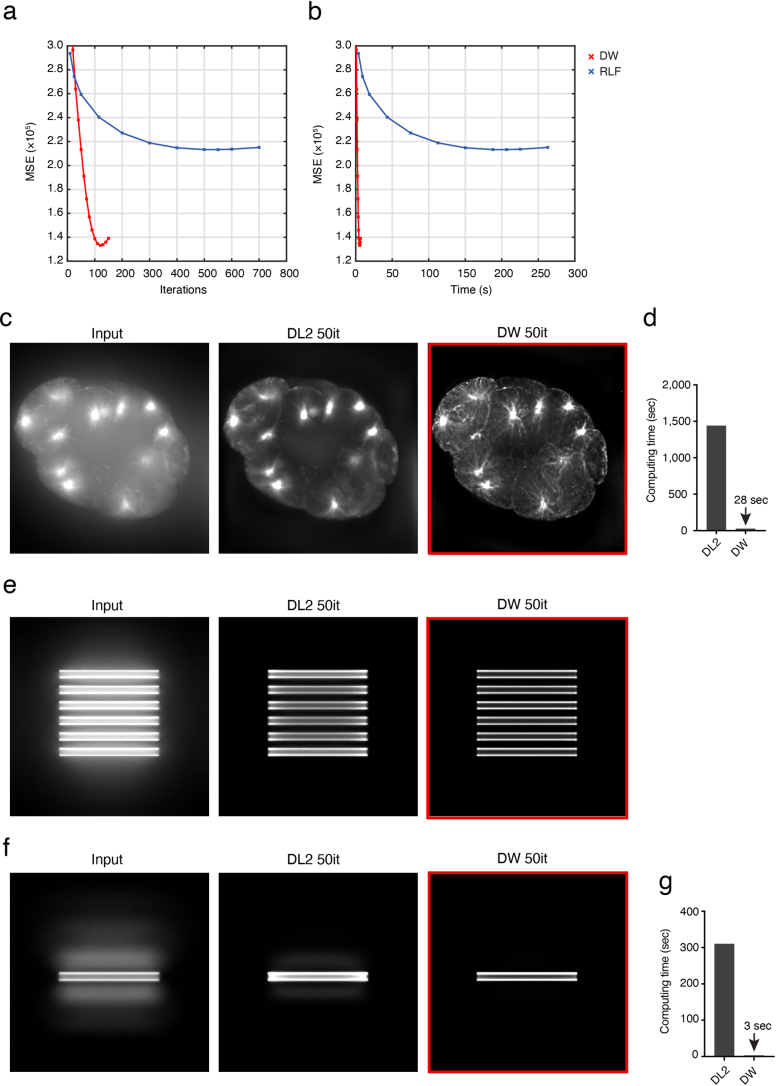
Deconwolf benchmarking. (**a, b**) Mean squared error (MSE) for the microtubule image shown in Fig. 1c deconvolved either by Deconwolf (DW) or by RedLionfish (RLF) depending on the number of iterations (a) or time of processing (b). Note that RLF did not manage to reach the same low MSE achieved by Deconwolf. (**c**) Maximum z-projection of a *C. elegans* whole-embryo z-stack image downloaded from http://bigwww.epfl.ch/deconvolution/bio/ before (Input) and after deconvolution using either DeconvolutionLab2 (DL2) (50 iterations, it) or Deconwolf. White, microtubules stained with falloidin. (**d**) Computing time required to generate the deconvolved images shown in (a) on an 8-Core AMD Ryzen 7 3700X machine. (**e**) Maximum z-projection of a synthetic hollow bar z-stack image downloaded from http://bigwww.epfl.ch/deconvolution/bars/ before (Input) and after deconvolution with DL2 or Deconwolf. The input image was corrupted by Gaussian noise and Poisson noise with a signal-to-noise ratio of 30 dB. The same dynamic range was used for all the images. (**f**) As in (c) but showing a single focal plane of the z-stack. (**g**) Computing time required to generate the deconvolved images shown in (c) and (d) on an 8-Core AMD Ryzen 7 3700X machine. Deconwolf-deconvolved images are framed in red. Source data

We first tested the performance of Deconwolf against Huygens Professional (v.17.04, Scientific Volume Imaging), a proprietary deconvolution software, and DeconvolutionLab2 (DL2, v.2.1.2)^16^, which are considered reference deconvolution tools. To this end, we leveraged a synthetic image of microtubules previously used to benchmark DL2 against Huygens Professional^16^. Using the Richardson–Lucy method implemented in Deconwolf (acceleration turned off) and comparing the deconvolved with the ground truth image, the smallest mean squared error (MSE) was 1.4 × 10^5^ at 1,500 iterations, whereas adding acceleration yielded the same MSE using only 115 iterations (Fig. 1b–d). We then used the same image to benchmark Deconwolf against DL2 and Huygens Professional. The sharpness and contrast of the Deconwolf-deconvolved image were superior compared with the same image processed by DL2 using the same number of iterations (115) or by Huygens Professional, as judged by eye as well as based on the MSE (1.4 × 10^5^, 2.3 × 10^5^ and 2.1 × 10^5^ for Deconwolf, DL2 and Huygens Professional, respectively) (Fig. 1e–g). Notably, performing the same number of iterations (115) with Deconwolf was more than 50-fold faster than using DL2 with the FFTW2 fast Fourier transform (FFT) library on an 8-Core AMD Ryzen 7 3700X machine. Reaching the same MSE achieved by Deconwolf (1.4 × 10^5^) by running DL2 on the same machine would require ~1,500 iterations and ~3 hours, whereas this took only 115 iterations and 16 second with Deconwolf: a 700-fold decrease in computing time. Accordingly, processing the same image with DL2 and the same settings previously used (500 iterations) required ~1 hour, while using Deconwolf we obtained a visual optimum with as little as 80 iterations in only 12 seconds (Fig. 1h,i). This dramatic difference in deconvolution speed mainly depends on the use of acceleration in Deconwolf; the use of the FFTW3 library^17^ in Deconwolf as opposed to the FFTW2 library in DL2; and the parallelization of all critical operations. Notably, although DL2 is compatible only with central processing units (CPUs), we dramatically increased the speed of Deconwolf by using an OpenCL GPU, reaching 80 iterations in 2 seconds using an AMD Radeon RX 6700 XT GPU (a 1,500-fold decrease in computing time). Of note, when we compared Deconwolf with RedLionfish (RLF, v0.9)^18^, another deconvolution software compatible with GPUs, Deconwolf was more than 100-fold faster than RLF on a standard laptop computer and, importantly, RLF did not reach the same low MSE achieved by Deconwolf (Extended Data Fig. 1a,b). Deconwolf also outperformed DL2 when applied to *Caenorhabditis elegans* whole-embryo and synthetic hollow bar images from the same source^16^ as the microtubule image described above (Extended Data Fig. 1c–g). These results show that combining SHB acceleration and FFTW3 in Deconwolf drastically improves the speed of deconvolution compared with the reference open-source deconvolution software DL2. Although using a GPU drastically increased the speed of Deconwolf, for practical reasons we performed all downstream computations using a CPU.

The second key feature of Deconwolf is the use of a high-precision PSF calculator (DW-PC) based on the Born–Wolf model^19^, which integrates over sensor pixels instead of sampling pixels only at their center, as in PSF Generator^20^, the gold-standard tool for PSF calculation (Methods and Supplementary Note 3). To benchmark DW-PC against PSF Generator, we used the PSFs generated by both tools as an input to Deconwolf (that is, varying how the PSF is generated and keeping other parameters constant) to deconvolve microscopy images previously generated by OligoFISSEQ^14^ (Supplementary Fig. 1a). In the images deconvolved using DW-PC, the fluorescent dots were more distinguishable in the (x,y) plane than the dots in the images deconvolved using PSF Generator (Fig. 1j,k and Supplementary Fig. 1b,c). Accordingly, the size of the fluorescent dots was significantly smaller in the DW-PC than in the PSF Generator images (full width at half maximum (FWHM), mean ± s.d., 439.9 ± 79.7 nm versus 477.4 ± 87.1 nm, *P* = 1.3 × 10^–29^, Wilcoxon test, two-tailed), improving the resolvability of OligoFISSEQ dots (Fig. 1l and Supplementary Fig. 1b,c).

**Fig. 2:**
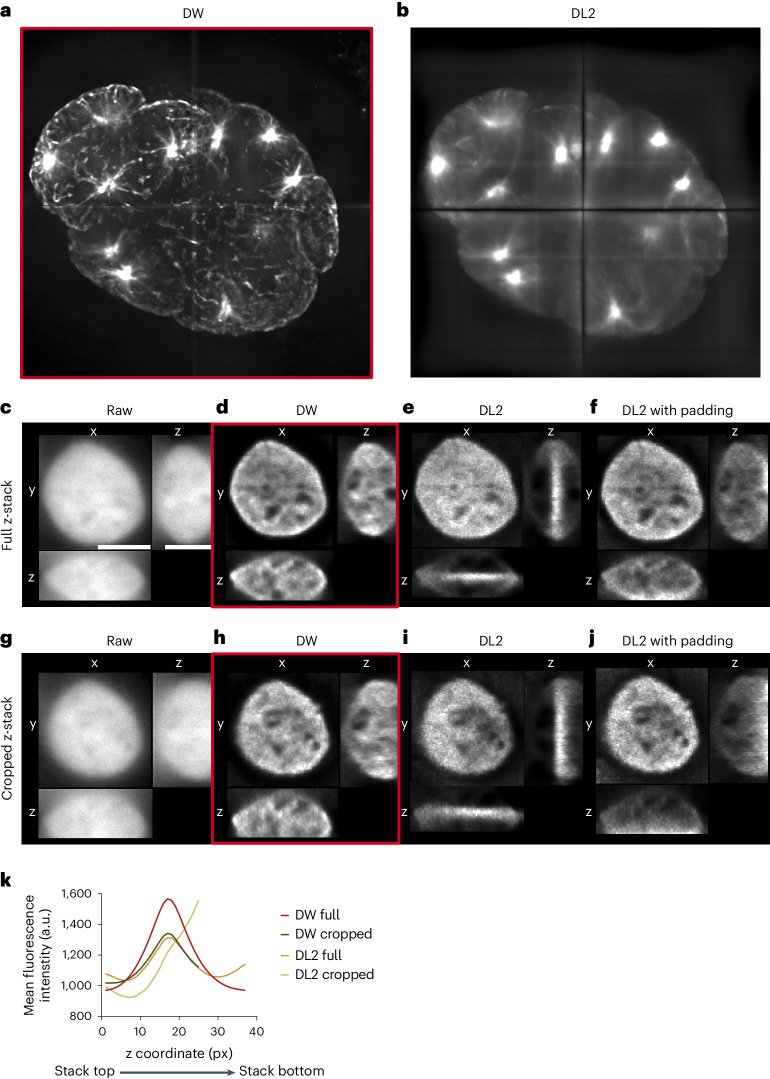
Deconwolf handles lateral and axial boundary effects. **a**,**b**, *C.* *elegans* whole-embryo image split into four cuboids and deconvolved with Deconwolf (DW) (**a**) or using the default option with no boundary handling in DeconvolutionLab2 (DL2) (**b**). **c**–**j**, x-, y- and z-projections of a human HAP1 cell nucleus (gray) without (raw) or after deconvolution with Deconwolf or DL2 (with or without padding) (**c**–**f**) and after removing the 12 bottom focal planes of the z-stack image (**g**–**j**). Imaging: widefield, ×100 oil objective (NA 1.45). **k**, Mean fluorescence intensity profile along the z-axis in **c**–**j**. The DL2 curves refer to deconvolution with uniform padding. Deconwolf-deconvolved images are framed in red. Micrographs in **c**–**i** are from a single experiment. A link to the Source Data for this figure is provided in the Data Availability statement. Source Data

**Extended Data Fig. 2:**
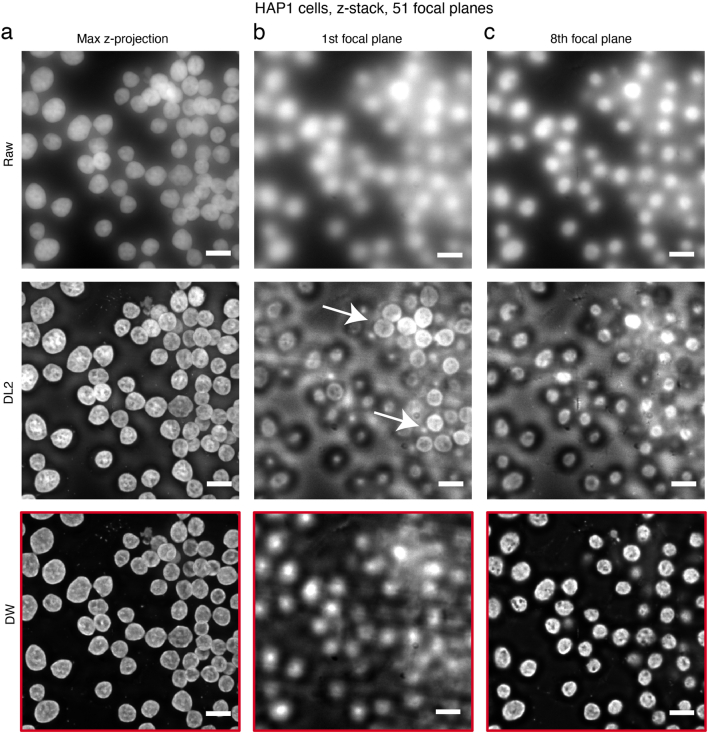
Deconwolf outperforms DeconvolutionLab2 in handling axial boundary effects. **a**) Maximum z-projection of a z-stack image with 51 focal planes from HAP1 human chronic myeloid leukemia cell nuclei stained with Hoechst 33342, before (Raw) or after deconvolution with DeconvolutionLab2 (DL2, no padding or apodization) or with Deconwolf (DW). (**b**) As in (a) but showing only the 1^st^ focal plane from the top of the z-stack. The white arrows indicate two examples of artifact generated by DL2, which are not visible in the corresponding Deconwolf-deconvolved image. (**c**) As in (a) but showing only the 8^th^ focal plane from the top of the z-stack. Scale bars, 10 μm. Deconwolf-deconvolved images are framed in red. All micrographs are from a single experiment.

The third key feature of Deconwolf is the implementation of a method for handling image boundary effects originally developed in astrophysics^21^. This approach considers the outside of an image as missing data in contrast to standard boundary handling methods, which either use an explicit guess of what is outside the imaged region (padding) or treat the image boundary circularly (with or without apodization) (Supplementary Note 4). To benchmark the boundary handling method implemented in Deconwolf (DW-BH), we first assessed how it performs in comparison with any of the five boundary handling methods implemented in DL2 (DL2-BH), using the same *C.* *elegans* whole-embryo image described above and the same number of iterations (50). The images deconvolved with DW-BH were much sharper and resulted in considerably fewer lateral boundary artifacts compared with the images processed using the default DL2-BH or any of the other boundary handling methods in DL2 (Fig. 2a,b and Supplementary Fig. 2). Next, we assessed the ability of DW-BH to handle boundary effects that may arise during the deconvolution of z-stacks, especially when an object is only partially imaged along the z-direction. To this end, we deconvolved z-stack images of human cell nuclei, showing that DL2-BH, but not DW-BH, introduces clearly visible distortions along the z-axis (Fig. 2c–f and Extended Data Fig. 2). Notably, these artifacts were only partially prevented by using the padding option in DL2 and were enhanced by cropping the bottom focal planes to mimic a frequent set-up in fluorescence microscopy experiments (Fig. 2f–j). Accordingly, although this had a modest effect on the fluorescence intensity profile along the z-axis in Deconwolf-deconvolved images, the same procedure drastically changed the z-profile of DL2-deconvolved images (Fig. 2k). Altogether, these results demonstrate that Deconwolf outperforms DL2 in terms of both the quality and fidelity of the deconvolved images generated, as well as in terms of the time required to generate them.

### Deconwolf does not generate artifactual signals

**Fig. 3:**
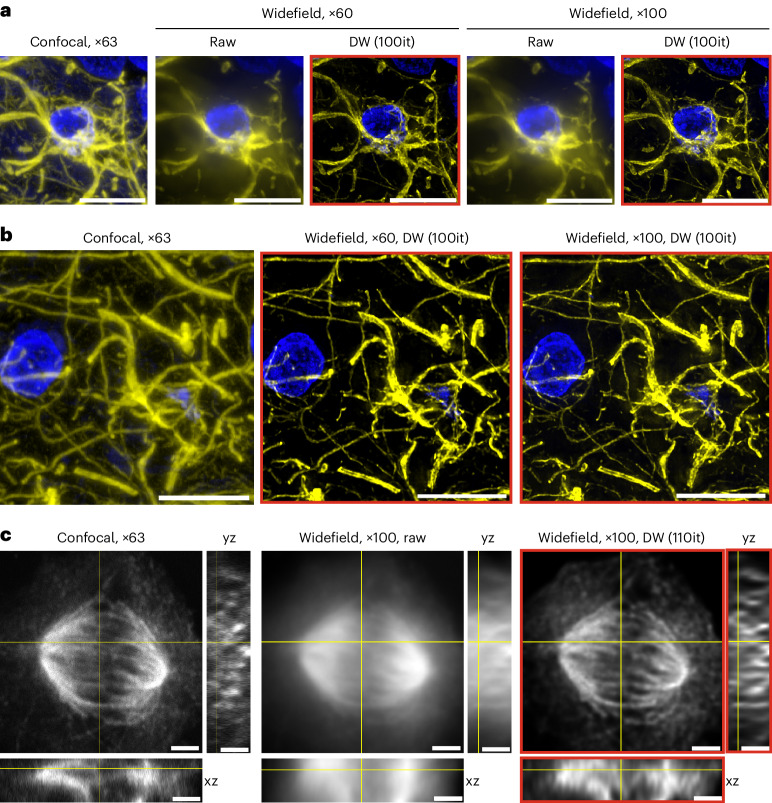
Widefield imaging combined with Deconwolf generates images of comparable quality compared with standard confocal imaging. **a**, Human brain tissue section stained with an antibody against GFAP (yellow) and imaged either on a confocal microscope with a ×63 oil objective or on a widefield microscope using either ×60 (NA = 1.40) or ×100 (NA = 1.45) oil objectives, with or without deconvolution with Deconwolf (DW). Maximum z-projections are shown. Blue, DNA. Scale bars, 10 μm. **b**, As in **a** for a different field of view. Scale bars, 10 μm. **c**, x–y views (large squares) and orthogonal views (x–z and y–z) of a single focal plane of a z-stack image from U-2 OS osteosarcoma cells undergoing immunofluorescence with an antibody against tubulin and imaged either on a confocal microscope using a ×63 oil objective or on a widefield microscope using a ×100 (NA = 1.45) oil objective, with or without deconvolution with Deconwolf. Scale bars, 2 μm. Deconwolf-deconvolved images are framed in red. All micrographs are from a single experiment. A link to the Source Data for this figure is provided in the Data Availability statement.

**Extended Data Fig. 3:**
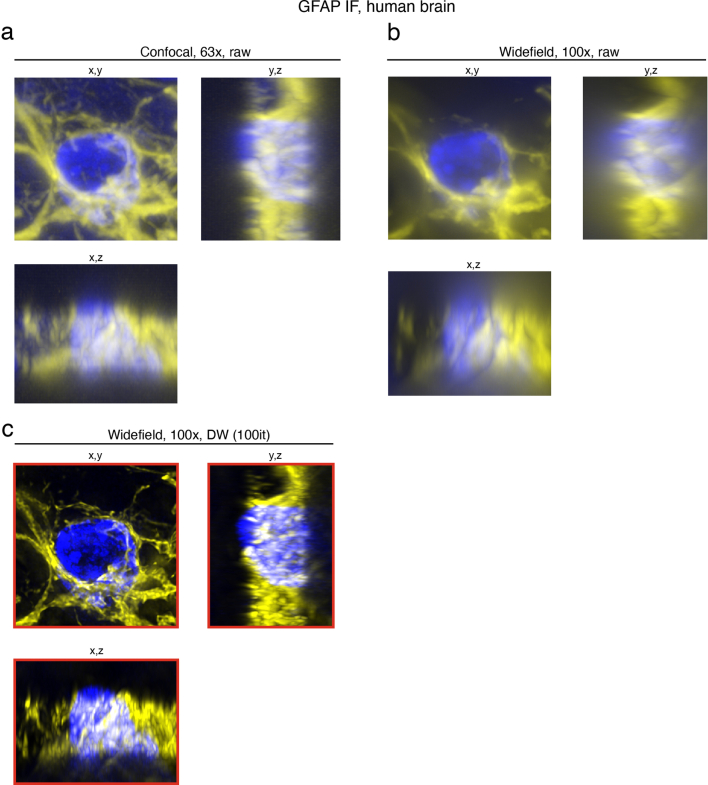
Deconwolf improves the quality of widefield images also in the axial dimension. (**a-c**) Maximum z-projections in both lateral and axial views of a z-stack image of a nucleus from a human brain tissue section stained with an antibody against the Glial Fibrillary Acidic Protein (GFAP) (yellow) and imaged either (a) on a confocal microscope with a ×63 oil objective or (b, c) on a widefield microscope using a ×100 oil objective (NA = 1.45), with or without deconvolution with Deconwolf (DW). Blue, DNA. Scale bars, 10 μm. Deconwolf-deconvolved images are framed in red. All micrographs are from a single experiment.

To further assess the reliability of Deconwolf, we sought to compare it with confocal microscopy, considering images generated with the latter as ground truth. To ensure that Deconwolf does not generate artifacts in the form of new signals absent in confocal images, we imaged the same region of a human brain tissue section stained with an antibody against glial fibrillary acidic protein (GFAP) using a widefield and confocal microscope (Methods). As expected, confocal images were sharper and had more structural details than the non-deconvolved widefield images acquired in the same tissue region using similar optical magnification (×60 for widefield and ×63 for confocal) (Fig. 3a,b and Extended Data Fig. 3). However, when we deconvolved the same widefield images using Deconwolf, the GFAP pattern became considerably sharper, and we could not detect any structures present in Deconwolf-deconvolved images that were not in the corresponding confocal ones (Fig. 3a,b and Extended Data Fig. 3). In contrast, DL2 did not manage to achieve the same quality as Deconwolf (Supplementary Fig. 3). Similar improvements were obtained using a different confocal microscope (Fig. 3c and Methods). We also applied Deconwolf to images obtained either by classical confocal microscopy or stimulated emission depletion (STED) super-resolution microscopy, showing that Deconwolf can be effectively used to enhance the sharpness of these images (Supplementary Fig. 4 and Methods). We note, however, that although the quality of Deconwolf-deconvolved confocal and STED images is clearly superior than the corresponding raw images, the latter show details in the low-intensity range that are not clearly visible in the corresponding deconvolved images. Hence, the choice between widefield and confocal imaging will depend on the resolution, speed and throughput required in a given application and on the available budget.

### Deconwolf enables transcript detection in crowded smFISH images

**Fig. 4:**
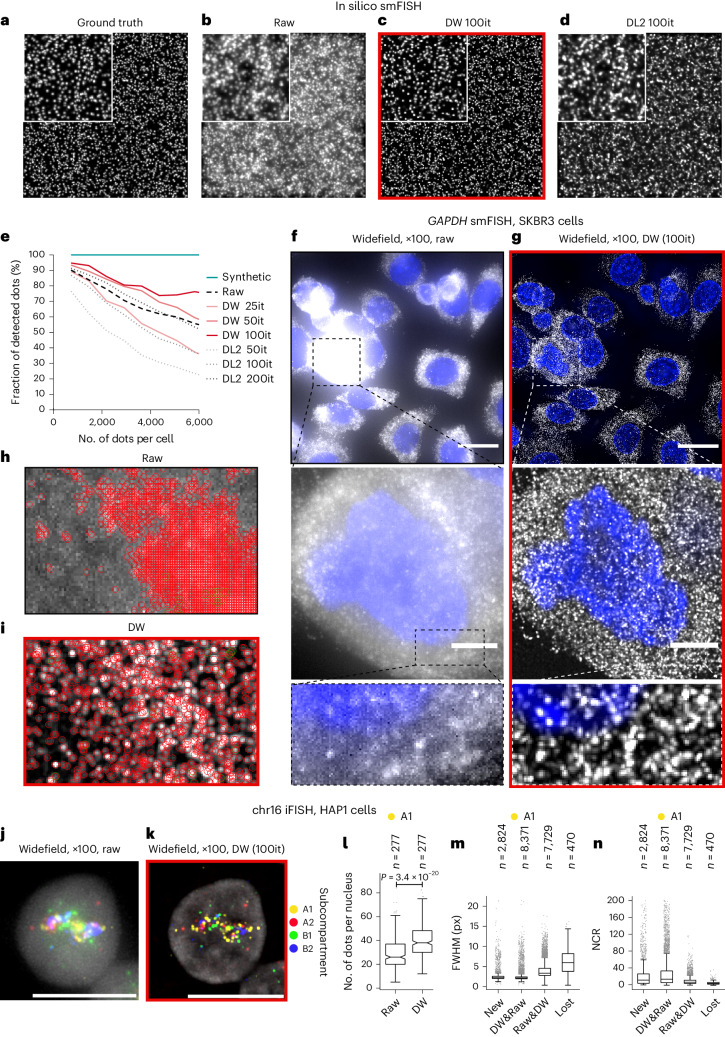
Deconwolf greatly improves signal detection in crowded FISH images. **a**–**d**, In silico smFISH. **a**, Maximum z-projection of an in silico generated z-stack containing high-density diffraction limited dots. **b**, As in **a** after applying noise to simulate a real smFISH image. **c**, As in **b** after applying Deconwolf (DW). **d**, As in **b** after deconvolution with DL2. **e**, Percentage of dots detected after deconvolution of in silico smFISH images. **f**,**g**, Human SKBR3 cells stained with smFISH probes targeting *GAPDH* transcripts (white) (**f**) and after deconvolution with DW (**g**). Imaging: widefield, ×100 oil objective (NA 1.45). Maximum z-projection is shown. Blue, DNA. Scale bars, 20 μm in top panel, 5 μm in middle panel. **h**, Zoom-in view of one field of view in **f** with *GAPDH* transcripts identified by DOTTER encircled in red. **i**, *GAPDH* transcripts identified by DOTTER in the same field of view as in **h** after deconvolution with Deconwolf. **j**,**k**, HAP1 cells stained with iFISH probes targeting 63 loci along chromosome 16 (**j**) and after deconvolution with Deconwolf (**k**). Imaging: widefield, ×100 oil objective. Maximum z-projection of one nucleus is shown. Gray, DNA. Scale bars, 10 μm. **l**, iFISH dot counts per nucleus for loci in the A1 chromatin subcompartment. *P* value, Wilcoxon test, two-tailed. *n*, number of cells. **m**, FWHM of the dots in **l**. New, dots detected only in Deconwolf-deconvolved images. Lost, dots detected in raw images but not after applying Deconwolf. DW&Raw, dots detected in Deconwolf-deconvolved images that are also detected in the corresponding raw images. Raw&DW, dots detected in raw images that are also detected in the corresponding Deconwolf-deconvolved images. *n*, number of dots. **n**, Nuclear–contrast ratio (NCR) values of the iFISH dots in **l**. Each boxplot extends from the 25th to the 75th percentile, horizontal bars represent the median, whiskers extend from –1.5 × IQR to +1.5 × IQR from the closest quartile. Gray dots, outliers. Deconwolf-deconvolved images are framed in red. All micrographs are from a single experiment. A link to the Source Data for this figure is provided in the Data Availability statement. Source Data

**Extended Data Fig. 4:**
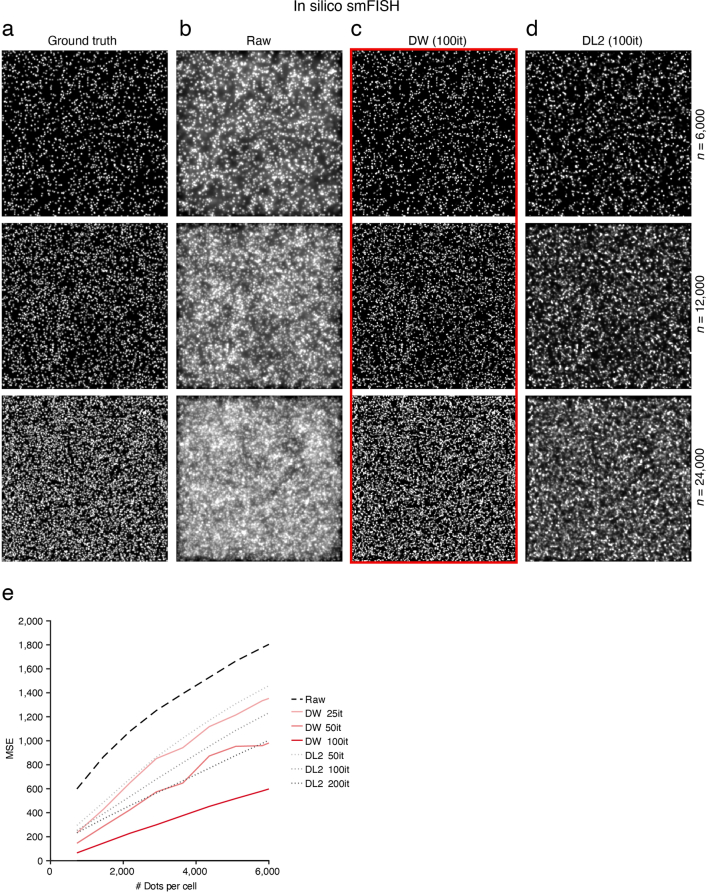
Deconwolf greatly improves the detection of diffraction limited dots in highly crowded images. (**a**) Maximum z-projections of z-stack images containing increasing numbers (*n*) of in silico generated diffraction limited dots. Only the 10 middle focal planes were used to make the images of the projections, while all the planes were used for the quantifications shown in (e). (**b**) As in (a) but after applying noise to simulate an image that would be obtained by applying smFISH to visualize the transcripts of a highly expressed gene. (**c**) As in (b) but after deconvolving the images with Deconwolf (DW) (100 iterations, it). (**d**) As in (c) but using DeconvolutionLab2 (DL2). (**e**) Mean squared error (MSE) of dot detection using in silico smFISH images containing a different number of dots, such as those shown in (b), after applying Deconwolf or DL2 with different numbers of iterations. Deconwolf-deconvolved images are framed in red. All micrographs are from a single experiment. Source data

We then explored whether Deconwolf could be used to increase the spatial resolution of FISH experiments, which often yield images with spatially crowded (near-)diffraction limited fluorescent dots. To this end, we first generated in silico images containing different densities of diffraction limited fluorescent dots and added a Poissonian and Gaussian noise component to simulate real smFISH images (Methods). Using these images as ground truth, we analyzed the ability of different deconvolution tools to identify true dots (Methods). Deconwolf again outperformed DL2 even when the number of iterations in DL2 was twice as high (Fig. 4a–e and Extended Data Fig. 4). To further assess the performance of Deconwolf, we varied the noise component (signal-to-noise ratio, 5–40) and simulated confocal images of smFISH dots in addition to widefield ones. In both cases, Deconwolf drastically improved the quality of the images (Supplementary Fig. 5a–j). In the case of widefield images, even for very noisy images (signal-to-noise ratio = 5) containing a relatively high number of dots (1,600 dots per z-stack, corresponding to ~500 dots in a 20-μm-diameter spherical cell), deconvolution with Deconwolf enabled identification of as many as 91% of the true dots (Supplementary Fig. 5k). In contrast, only ~29% of the dots in the ground truth images could be identified without Deconwolf (Supplementary Fig. 5k). This corresponds to a threefold improvement in the detection sensitivity for Deconwolf-deconvolved images for some of the conditions tested (Supplementary Fig. 5l). For more realistic signal-to-noise ratio values typically encountered in smFISH experiments (signal-to-noise ratio = 40), Deconwolf applied to widefield images achieved high detection sensitivity even at very high densities (Supplementary Fig. 5k). Similarly, the dot detection efficiency was drastically improved in Deconwolf-deconvolved confocal images compared with non-deconvolved images (Supplementary Fig. 5m,n). Comparing confocal with widefield Deconwolf-deconvolved images, the dot detection efficiency was substantially higher in the latter in most of the cases (Supplementary Fig. 6).

**Extended Data Fig. 5:**
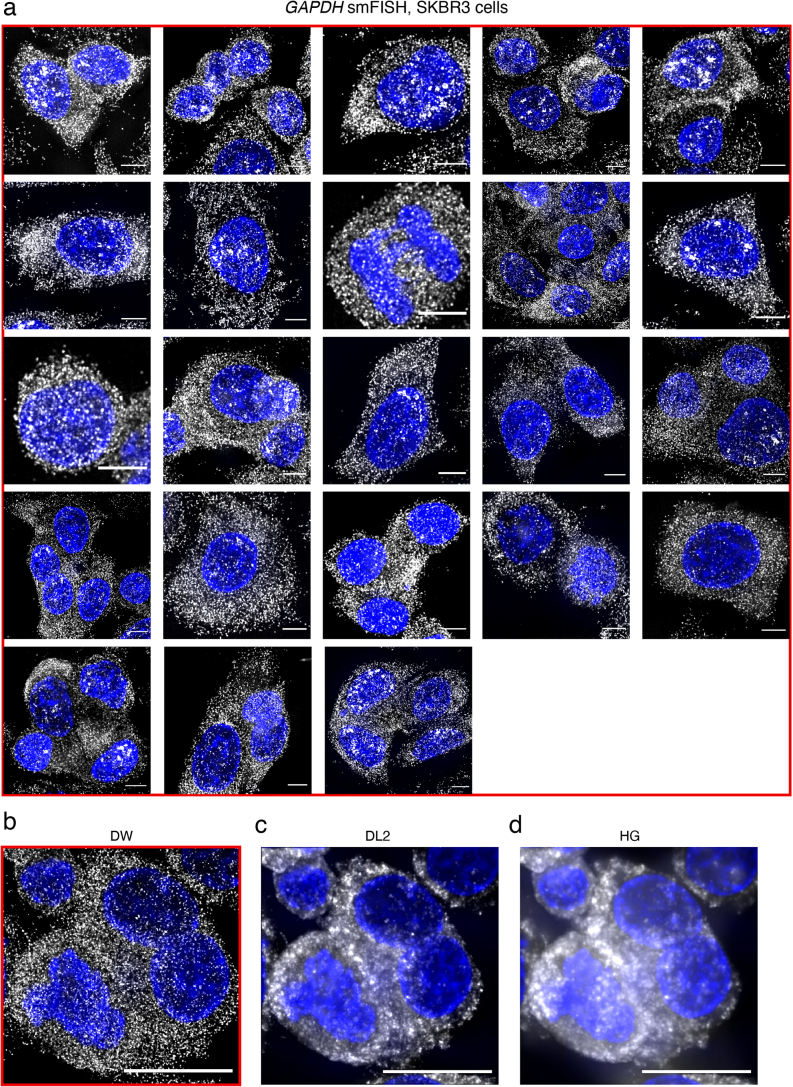
Deconwolf drastically improves the visualization of individual transcripts in crowded smFISH images. (**a**) Maximum z-projections of multiple fields of view of SKBR3 human breast carcinoma cells stained with an smFISH probe targeting *GAPDH* gene transcripts (white) and imaged on a widefield microscope using a ×100 objective, after deconvolution with Deconwolf. Blue, DNA stained with Hoechst 33342. Scale bars, 5 μm. (**b**–**d**) Maximum z-projection of one field of view of SKBR3 human breast carcinoma cells stained with an smFISH probe targeting GAPDH gene transcripts (white) and imaged on a widefield microscope using a ×100 objective, after deconvolution with Deconwolf (DW) (b), DeconvolutionLab2 (DL2) (c) or Huygens Professional (HG) (d). Blue, DNA stained with Hoechst 33342. Scale bars, 20 μm. Deconwolf-deconvolved images are framed in red. All micrographs are from a single experiment.

**Extended Data Fig. 6:**
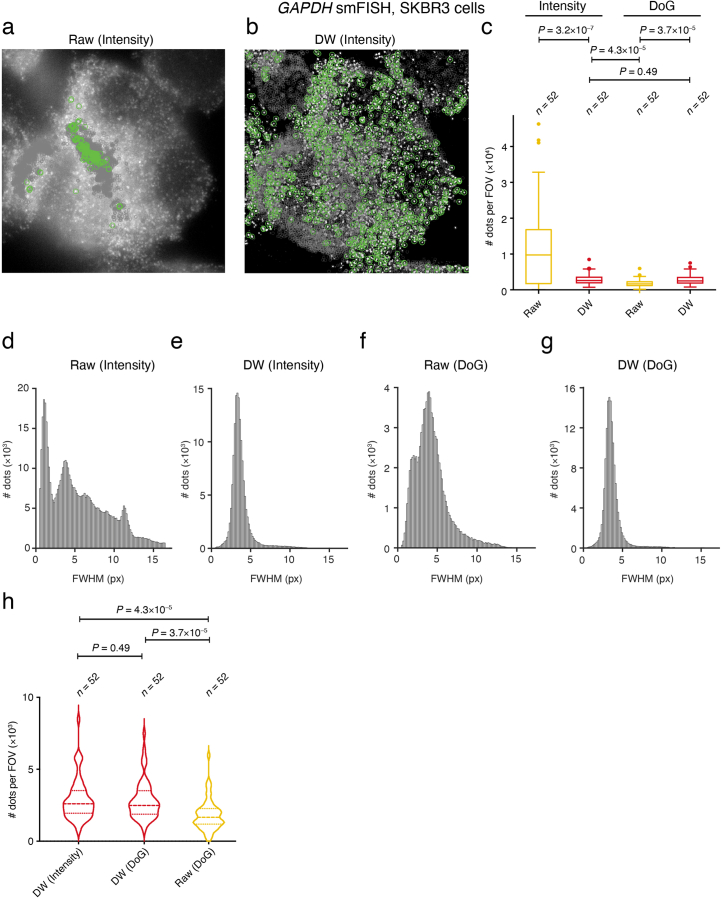
Deconwolf drastically improves the sensitivity and specificity of dot detection in crowded smFISH images. (**a, b**) Maximum z-projection of a z-stack image from SKBR3 human breast carcinoma cells subjected to smFISH with a probe for *GAPDH* transcripts (white) and imaged on a widefield microscope using a ×100 oil objective, without (Raw) (a) or with deconvolution with Deconwolf (DW) (b). For clarity, only *GAPDH* transcripts detected in a single focal plane are shown in green. Individual transcripts were identified using the intensity-based dot detection module (Intensity) in DOTTER. First, we manually selected a threshold in DOTTER to identify what visually appeared as ‘true’ smFISH signals in the deconvolved z-stack (dots encircled in green in (b)). We then asked DOTTER to find the same number of dots in the corresponding non-deconvolved z-stack, choosing from the brightest dots automatically identified by DOTTER (dots encircled in green in (a)). (**c**) Distributions of the number of *GAPDH* gene transcripts identified in the images shown in Supplementary Fig. 7, with or without deconvolution with Deconwolf, either with the intensity-based (Intensity) or with the Difference of Gaussians (DoG) dot detection module in DOTTER. FOV, field of view. *n*, number of FOVs analyzed. *P*, Wilcoxon matched-pair signed rank test, two-tailed. Each boxplot extends from the 25^th^ to the 75^th^ percentile, the horizontal bar represents the median, and whiskers extend from –1.5×IQR to +1.5×IQR from the closest quartile, where IQR is the inter-quartile range. Dots, outliers. (**d-g**) Distributions of full width at half maximum (FWHM) values of the same smFISH dots analyzed in (c). (**h**) As in (c) but displaying the distributions with violin plots and skipping the ‘Raw (Intensity)’ dot group. *n*, number of FOVs analyzed. Each violin plot extends from minimum to maximum. The horizontal dashed line represents the median, while the bottom and upper dotted horizontal lines indicate the first and third quartile, respectively. Micrographs in (a-b) are from a single experiment. Source data

Next, we assessed whether Deconwolf would also help resolve crowded transcripts in real smFISH images acquired with a widefield microscope. To this end, we performed smFISH with a probe targeting *GAPDH* transcripts in human SKBR3 cells (Supplementary Table 1 and Methods). As expected, the *GAPDH* gene was expressed at very high levels in most of the cells, and the corresponding smFISH dots were often too crowded to be resolved by eye in non-deconvolved (raw) images (Fig. 4f). However, when we deconvolved the images with Deconwolf, but not with DL2 or Huygens Professional, the resolution drastically improved, making individual dots visible even in very crowded regions (Fig. 4g, Supplementary Fig. 7 and Extended Data Fig. 5a–d). To quantitatively assess the performance of Deconwolf for these crowded images, we applied our in-house software DOTTER (v0.598), which is tailored to detect diffraction limited dots in FISH images (Methods). In Deconwolf-deconvolved smFISH images, DOTTER managed to detect individual transcripts simply based on fluorescence intensity, whereas this was not possible in the original (raw) images. Instead, a significantly higher number of dots (*P* = 3.16 × 10^–7^, *t*-test, two-sided) was detected in raw images, especially in very bright regions (Fig. 4h,i and Extended Data Fig. 6a–c). The high local concentration of dots in these regions results in high levels of blurred or out-of-focus light, elevating nearby dim signals and hence hampering the distinction between true-positive and false-positive dots. The dots detected using DOTTER in Deconwolf-deconvolved images had a narrow size distribution, which is characteristic of smFISH signals^12^, whereas the dots identified in the corresponding raw images had a broader size distribution, suggesting that many of those dots represent false-positive signals (Extended Data Fig. 6d,e).

To confirm the ability of Deconwolf to resolve smFISH signals in crowded images, we used a different dot detection procedure (difference of Gaussians or DoG), which is also implemented in DOTTER (Methods). In this case, the number of dots detected using DOTTER was significantly lower (*P* = 3.66 × 10^–5^, *t*-test, two-sided) in the raw images compared with Deconwolf-deconvolved ones (Extended Data Fig. 6c). Notably, both the intensity-based and DoG-based approaches yielded very similar dot counts in the case of deconvolved images (Extended Data Fig. 6c). Furthermore, the size distribution of the dots detected using the DoG approach was still considerably broader in the raw images compared with the same images after deconvolution, suggesting that many of the dots detected in raw images represent false-positive signals (Extended Data Fig. 6f,g). Accordingly, the corresponding distribution of dot counts per field of view was significantly different in the case of raw images analyzed using the DoG approach compared with Deconwolf-deconvolved images analyzed with any of the two dot detection approaches (Extended Data Fig. 6h). These results further showcase the robustness of Deconwolf and demonstrate that Deconwolf can dramatically improve the sensitivity and specificity of smFISH.

### Deconwolf resolves densely packed DNA loci in DNA FISH images

**Extended Data Fig. 7:**
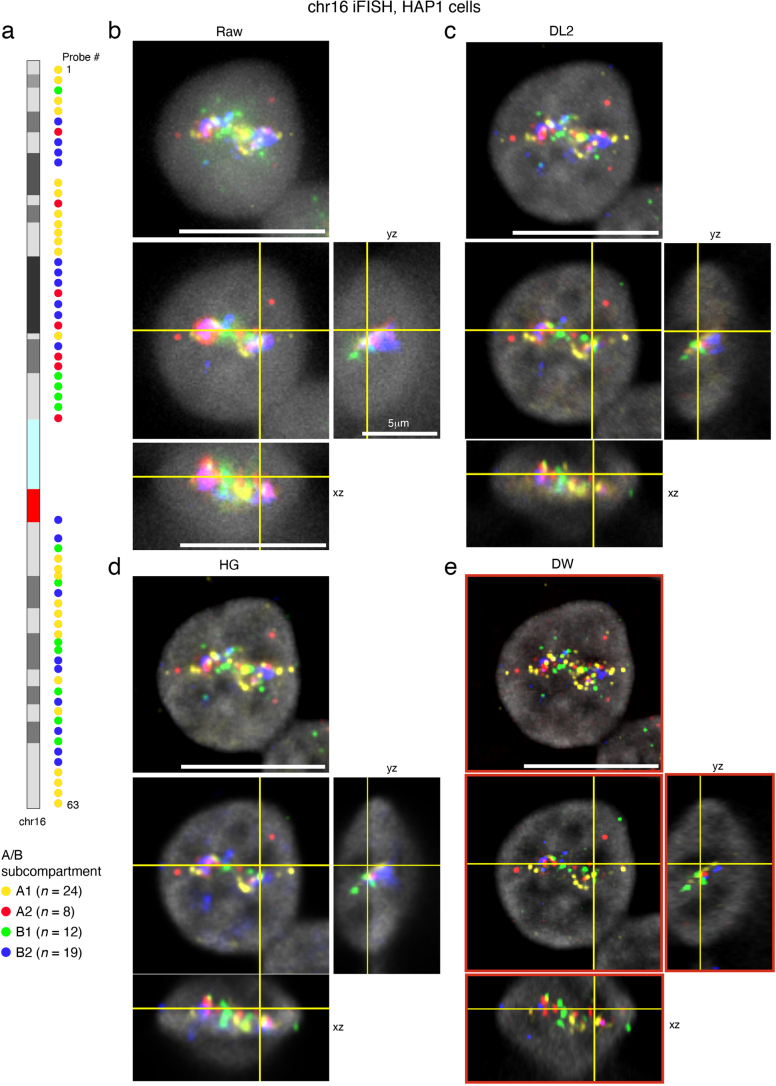
Deconwolf improves the sensitivity and specificity of dot detection in iFISH images. (**a**) Location of the 63 iFISH probes used in this study to visualize 63 DNA loci belonging to different chromatin A/B subcompartments defined based on Hi-C, all along chr16. Different colors indicate the A/B chromatin subcompartment, in which the genomic locus targeted by each probe is located. The location of each probe is shown along the ideogram of chr16. Probes are labeled in ascending number from top to bottom. *n*, number of probes in each subcompartment. (**b**) Top: maximum z-projection of a z-stack image of a nucleus of HAP1 human myeloid leukemia cells subjected to iFISH with the 63 probes (colored dots) shown in (a) and imaged on a widefield microscope using a ×100 objective. Bottom: xy view (large square) as well as orthogonal views (xz and yz) of a single focal plane of the same z-stack image shown above. Gray, DNA stained with Hoechst 33342. Scale bars, 10 μm except in the yz view (5 μm). (**c–e**) As in (b) but after deconvolution with DeconvolutionLab2 (DL2) (c), Huygens Professional (HG) (d) and Deconwolf (DW) (e). All images were upscaled to a pixel size of 23 nm. Deconwolf-deconvolved images are framed in red. All micrographs are from a single experiment.

Next, we assessed the performance of Deconwolf on crowded images of DNA loci visualized using DNA FISH. To this end, we used the iFISH pipeline that we previously established^13^ to simultaneously visualize 63 DNA loci in different A or B chromatin subcompartments^22^ along chromosome 16 (Extended Data Fig. 7a, Supplementary Table 2 and Methods). In raw images or in images deconvolved with DL2 or Huygens Professional, the iFISH signals appeared as clouds of poorly distinguishable fluorescent dots inside the cell nucleus (Fig. 4j and Extended Data Fig. 7b–d). By contrast, after deconvolution with Deconwolf, the dots became clearly separated and, as a result, the dot counts per nucleus significantly increased close to the expected number (Fig. 4k,l, Extended Data Fig. 7e and Supplementary Fig. 8a–c). Notably, deconvolution removed large and poorly contrasted dots, while new dots with an FWHM close to the mean (representing bona fide iFISH signals) became apparent, given that they were not detected in non-deconvolved raw images (Fig. 4m,n and Supplementary Fig. 8d–i). These results show that Deconwolf can drastically improve the specificity and spatial resolution of DNA FISH.

### Deconwolf enables high-throughput smFISH in tissue sections

**Fig. 5:**
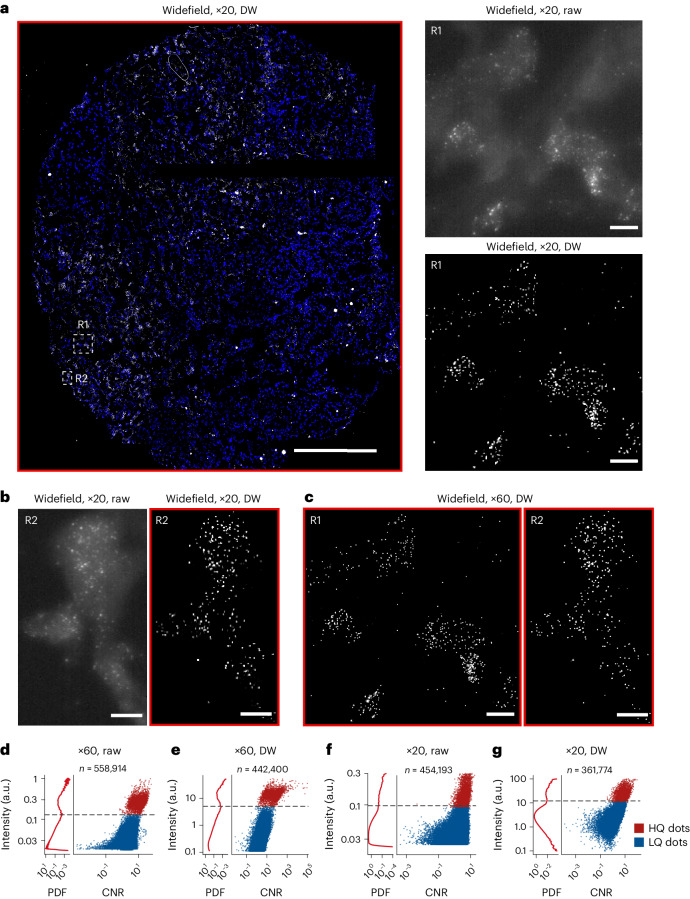
Deconwolf enables robust detection of individual transcripts in low-magnification smFISH images. **a**, Breast adenocarcinoma tissue section stained with an smFISH probe targeting *MKI67* transcripts (white) and imaged on a widefield microscope using a ×20 air objective, after deconvolution with Deconwolf (DW). Maximum z-projection is shown. Blue, DNA. Scale bars, 200 μm in the large left panel; 10 μm in the two small panels on the right. **b**,**c**, Zoom-in of the regions (R1 and R2) marked by the white dashed squares in **a**. Scale bars, 10 μm. **d**, Left plot: probability density function (PDF) of smFISH dot fluorescence intensities in one of five fields of view (FOV1) in **a** imaged at ×60 magnification (NA = 1.4). The dashed black line marks the intensity threshold that was used to separate high-quality (HQ) and low-quality (LQ) dots in the scatter plot. CNR, contrast-to-noise ratio. *n*, number of dots. **e**, As in **d** after applying Deconwolf. **f**, As in **d** after imaging the same field of view with a ×20 air objective (NA = 0.75). **g**, As in **f** after deconvolution with Deconwolf. Deconwolf-deconvolved images are framed in red. All micrographs are from a single experiment. A link to the Source Data for this figure is provided in the Data Availability statement.

**Extended Data Fig. 8:**
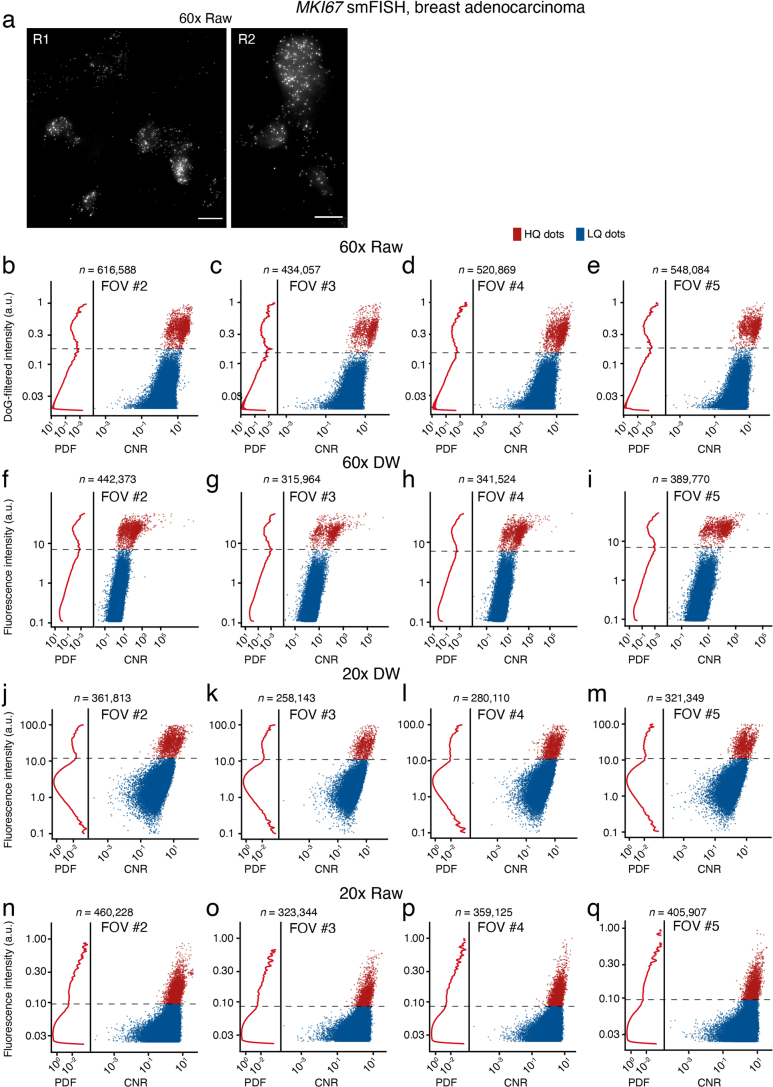
Deconwolf enables the detection of individual transcripts in low-magnification smFISH images. (**a**) Maximum z-projection of a z-stack image of the R1 and R2 regions shown in Fig. 5a imaged at ×60 magnification (oil objective). Scale bars, 10 μm. (**b-e**) In each panel, the left plot shows the probability density function (PDF) of the fluorescence intensity of the smFISH dots detected in the indicated field of view (FOV) imaged at ×60 magnification in the same tissue microarray core shown in Fig. 5a, using the DoG-based dot detection module in DOTTER. The local minimum marked by the dashed black line in the PDF plot was used to set a threshold in the DoG intensity to separate between high-quality (HQ) and low-quality (LQ) dots in the scatter plot on the right. CNR, contrast-to-noise ratio. *n*, number of dots analyzed. (**f-i**) As in (b-e) after deconvolving the corresponding images with Deconwolf (DW). (**j-m**) As in (f-i) but imaging the same FOVs with a ×20 magnification air objective and deconvolving the images with Deconwolf. (**n-q**) As in (j-m) but without deconvolution. Micrographs in (a) are from a single experiment.

Having demonstrated the ability of Deconwolf to resolve crowded (near-)diffraction limited dots in FISH images, we then wondered whether it would also enable the detection of individual transcripts in smFISH images acquired at low magnification (×20 air objective, NA = 0.75). To test this, we targeted the mRNA product of the *MKI67* gene in a tissue microarray and imaged an entire section of a breast carcinoma tissue core on a widefield microscope (Supplementary Table 1 and Methods). As expected, individual transcripts were poorly distinguishable in non-deconvolved (raw) images acquired at ×20 magnification (Fig. 5a,b). However, when we deconvolved the same images using Deconwolf the contrast improved dramatically, making individual *MKI67* mRNA molecules visible throughout the entire core (Fig. 5a,b). Importantly, the same spatial patterns of *MKI67* transcripts observed in ×20 deconvolved images were recapitulated in both raw and deconvolved images from the same fields of view acquired at higher magnification (×60 oil objective, NA = 1.4) (Fig. 5c and Extended Data Fig. 8a).

**Extended Data Fig. 9:**
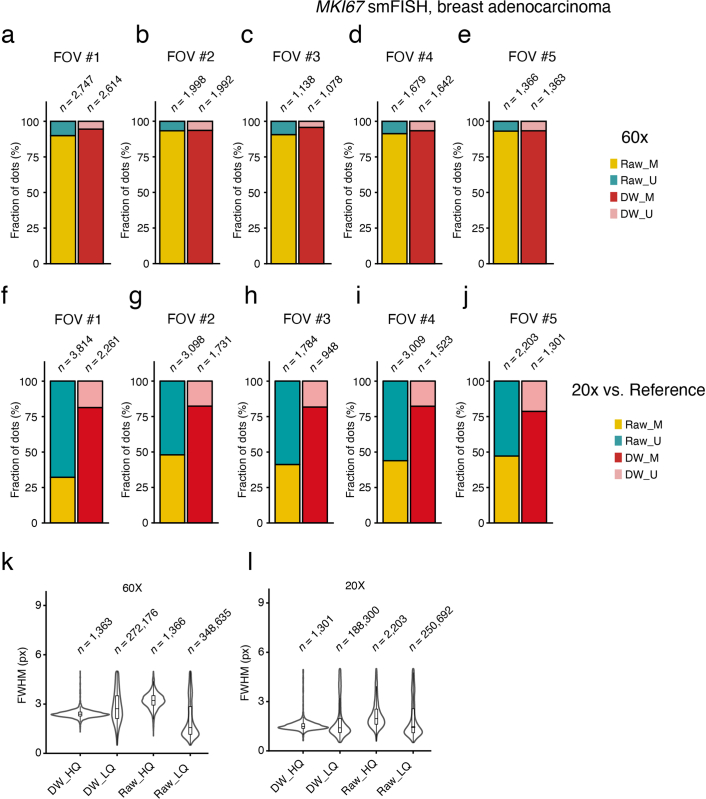
Deconwolf enables the detection of individual transcripts in low-magnification smFISH images. (**a-e**) Fraction of high-quality (HQ) dots from the scatter plots shown in Fig. 5d, e and Extended Data Fig. 8b–i that were matched (M) or unmatched (U) between corresponding raw and Deconwolf (DW)-deconvolved fields of view. FOV, field of view. Raw_M, dots identified in raw images that were also found in the corresponding deconvolved images. DW_M, dots identified in Deconwolf-deconvolved images that were also found in the corresponding raw images. (**f-j**) As in (a-e) but comparing the dots identified in images acquired at ×20 magnification with the dots shared by raw and Deconwolf-deconvolved images at ×60 magnification considering the latter as reference. (**k**) Distributions of the full width at half maximum (FWHM) values of HQ and LQ dots shown in the scatter plots in Fig. 5d, e and Extended Data Fig. 8b–i. *n*, number of dots. (**l**) As in (k) but for the HQ and LQ dots shown in the scatter plots in Fig. 5f, g and in Extended Data Fig. 8j–q. Violins extend from minimum to maximum, boxplots extend from the 25th to the 75th percentile, horizontal bars represent the median, whiskers extend from –1.5 × IQR to +1.5 × IQR from the closest quartile, where IQR is the inter-quartile range.

We then assessed whether robust automatic detection and counting of individual transcripts would be feasible in images acquired using a ×20 air objective. We first applied DOTTER to the same *MKI67* images but did not manage to automatically identify a reliable threshold for distinguishing real signals from noise. We therefore devised a different approach by calculating a contrast-to-noise ratio (CNR) and plotting it against the intensity of hundreds of thousands of fluorescent dots identified by DOTTER in the images (Methods). Except for non-deconvolved (raw) ×20 images, this approach identified two clearly distinct point clouds: one corresponding to high-quality smFISH dots with high CNR and intensity (most probably representing true signals), and the other corresponding to low-quality smFISH dots with lower intensity and low-to-intermediate CNR (most probably representing noise) (Fig. 5d–g and Extended Data Fig. 8b–e). In all of the images, except for the ×20 raw ones, the boundary between the two clouds corresponded to a local minimum clearly visible in the density plots of the fluorescence intensity of the DoG-filtered dots (Fig. 5d–g and Extended Data Fig. 8b–q). We therefore used this local minimum to set a threshold to automatically identify high-quality dots in ×60 as well as in ×20 deconvolved images. For the ×20 raw images, we selected the lower-density tail of the point cloud as containing high-quality dots (Fig. 5g and Extended Data Fig. 8n–q). In five fields of view analyzed, 91.6% of the high-quality dots identified in the raw images at ×60 magnification (NA = 1.4) matched the high-quality dots found in the corresponding deconvolved images (Extended Data Fig. 9a–e). Conversely, 94.1% of all of the high-quality dots identified in the deconvolved images overlapped with the high-quality dots in the corresponding raw images, suggesting that these represent true-positive signals (Extended Data Fig. 9a–e). We then used the high-quality dots shared between the raw and deconvolved ×60 images as the reference. A total of 58.3% of the high-quality dots (*n* = 8,105) in the ×20 raw images did not match the reference dots, suggesting that they represent false-positive signals (Extended Data Fig. 9f–j). In contrast, 81.3% of the high-quality dots (*n* = 6,313) identified in the ×20 deconvolved images matched the reference dots (Extended Data Fig. 9f–j). Of note, the high-quality dots identified in the deconvolved images had the narrowest size distribution, further suggesting that they represent true-positive signals (Extended Data Fig. 9k,l). These results demonstrate that widefield imaging with low-magnification objectives followed by Deconwolf can be used to reliably count smFISH dots across large tissue sections.

### Deconwolf improves the sensitivity of ISST

**Fig. 6:**
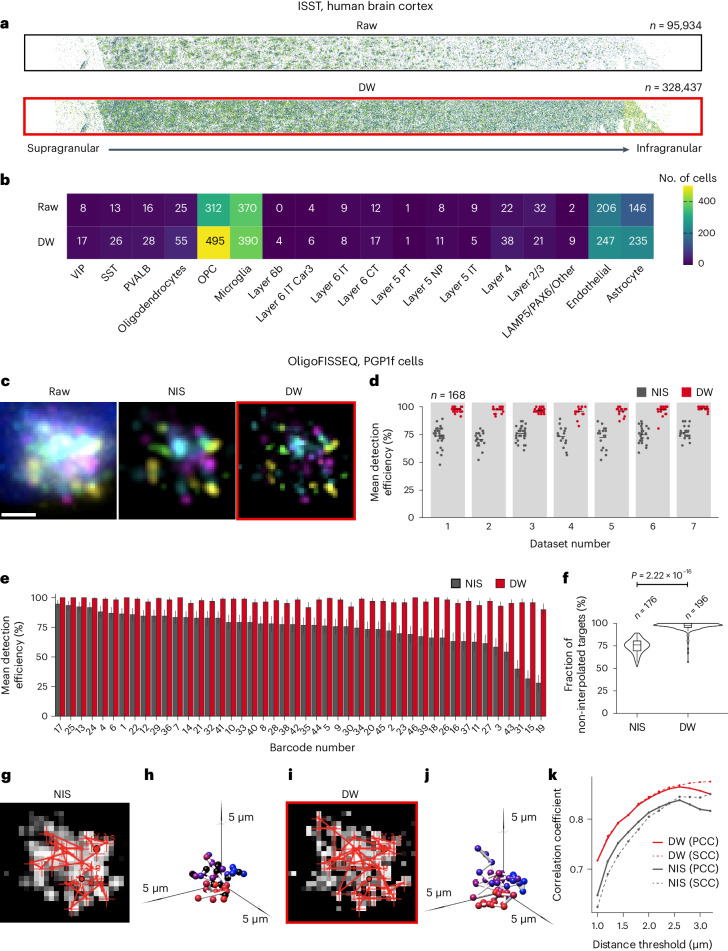
Deconwolf improves the sensitivity of ISST and accuracy of OligoFISSEQ*.* **a**, Spatial distribution of ISST dots identified in an ISST 120-gene dataset from human middle temporal gyrus cortex^23^, without (Raw) or after deconvolution with Deconwolf (DW). Each dot corresponds to an individual transcript. *n*, number of dots identified. **b**, Number of cells assigned to each of the 18 brain cell types shown along the x-axis. The nomenclature is the same as in ref. ^29^. CT, layer 6 corticothalamic neurons; IT, intratelencephalic neurons; NP, near-projecting neurons; OPC, oligodendrocyte precursor cells; PVALB, parvalbumin-expressing neurons; SST, somatostatin-expressing neurons; VIP, vasoactive intestinal peptide-expressing neurons. **c**, Maximum z-projection of a z-stack from the previously published ChrX-46plex OligoFISSEQ dataset^14^, without deconvolution (Raw) or after deconvolution with DW or with the deconvolution module in the commercial software NIS Elements AR (Nikon) (NIS). Scale bar, 2.5 μm. **d**, Detection efficiency in seven ChrX-46plex datasets from replicate experiments. Each dot represents one cell. *n*, total number of cells. Horizontal bars, mean values. **e**, Mean detection efficiency of each of the 46 OligoFISSEQ barcodes in the seven datasets in **b** after deconvolution with NIS or DW. Each bar indicates the fraction of positive observations over the total (*n*). Error bars, confidence interval calculated on 1,000 bootstrap replicates. **f**, Fraction of targets detected in X chromosome traces for which interpolation was not needed. *P* value, Wilcoxon’s test, two-tailed. *n*, number of X chromosome traces analyzed. Violins extend from minimum to maximum, boxplots extend from the 25th to the 75th percentile, horizontal bars represent the median, whiskers extend from –1.5 × IQR to +1.5 × IQR from the closest quartile. **g**, Example of chromatin fiber tracing in one nucleus from the OligoFISSEQ datasets shown in **b**, after deconvolution with the NIS software. Red segments represent connections between consecutive loci on the X chromosome. **h**, Rendering of the chromatin path shown in red in **g**. **i**, Same as in **g** but after deconvolution of the image with Deconwolf. **j**, Rendering of the chromatin path shown in red in **h**. **k**, Pearson’s (PCC) and Spearman’s (SCC) correlation coefficients of the correlation between inter-loci contact frequencies measured by OligoFISSEQ and Hi-C for the 46 consecutive DNA loci in the ChrX-46plex OligoFISSEQ dataset^14^, at different distance thresholds. Deconwolf-deconvolved images are framed in red. A link to the Source Data for this figure is provided in the Data Availability statement. Source Data

**Extended Data Fig. 10:**
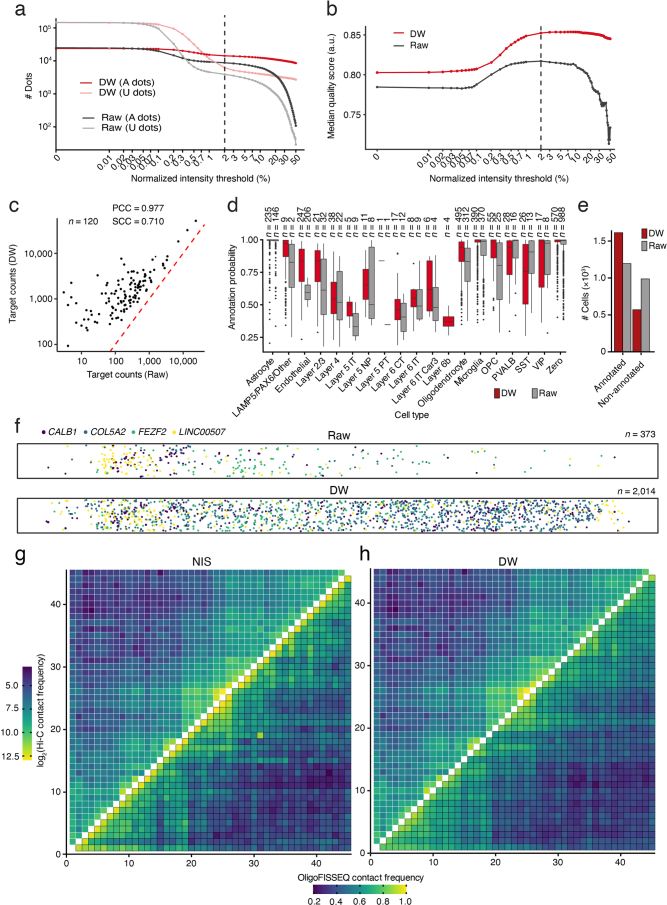
Deconwolf considerably improves the sensitivity of in situ spatial transcriptomics (ISST). (**a**) Absolute dot counts at different normalized intensity thresholds before (Raw) and after deconvolution with Deconwolf (DW), using images from a previously described ISST dataset consisting of 120 different genes imaged in a tissue section of human middle temporal gyrus cortex (see ref. ^23^). A, assigned dots representing bona fide individual transcripts identified based on the same pipeline previously used to process the same ISST dataset. U, dots with improper barcodes and thus discarded by the same pipeline. The vertical dashed black line represents the threshold used for all subsequent analyses of this dataset. (**b**) Median quality score of the A dots shown in (a), calculated at different fluorescence intensity thresholds as described in ref. ^23^. The vertical dashed black line represents the threshold used for all subsequent analyses of this dataset. (**c**) Scatter plot showing the correlation between the number of transcripts identified in images from a previously described ISST dataset (see ref. ^23^) consisting of 120 different genes (*n*) imaged in a tissue section of human middle temporal gyrus (MTG) cortex, before (Raw) and after deconvolution with Deconwolf (DW). Each dot represents one of the 120 genes analyzed. PCC, Pearson’s correlation coefficient. SCC, Spearman’s correlation coefficient. The dashed red line represents the bisector (that is, if transcript counts were equal in Raw and DW images, the dots should be aligned along this line). (**d**) Distribution of the annotation probability for each of the 120 gene transcripts profiled by ISST, in non-deconvolved (Raw) images or in Deconwolf-deconvolved images. Zero indicates cells that could not be annotated. The cell type nomenclature is as in ref. ^29^. CT, corticothalamic. IT, intratelencephalic. NP, near-projecting. VIP, vasoactive intestinal peptide expressing neurons. SST, somatostatin expressing neurons. PVALB, parvalbumin expressing neurons. OPC, oligodendrocyte precursor cells. *n*, number of cells in each group. Both DW and Raw counts sum up to the same total number of cells (*n* = 2,183). Each boxplot extends from the 25^th^ to the 75^th^ percentile, the horizontal bar represents the median, and whiskers extend from –1.5 × IQR to +1.5 × IQR from the closest quartile, where IQR is the inter-quartile range. Black dots, outliers. (**e**) Number of cells that could or could not be assigned to any of the 18 brain cell types shown in (b) using either non-deconvolved (Raw) images or Deconwolf-deconvolved images. (**f**) Spatial distribution of the ISST dots identified in the same MTG cortical stripe schematically shown in Fig. 6a for five layer-specific genes, without (Raw) or with deconvolution using Deconwolf. Each dot corresponds to an individual transcript. Left to right: supragranular to infragranular. *n*, number of dots identified. (**g**) Contact frequency maps for the 46 DNA loci on chrX in the ChrX-46plex OligoFISSEQ dataset described in ref. ^14^. Contact frequencies were calculated based on Hi-C data (top-left triangle) or OligoFISSEQ images deconvolved with the NIS Elements AR (Nikon) software (NIS, bottom-right triangle). (**h**) Same as in (g) but using Deconwolf (DW)-deconvolved OligoFISSEQ images.

Having demonstrated that Deconwolf drastically improves dot detection in both crowded and low-magnification FISH images, we wondered whether it could also improve signal detection in images generated by ISST. To this end, we applied Deconwolf to a 120-gene image dataset previously generated by applying ISST to a large section of the human middle temporal gyrus cortex^23^ (Supplementary Table 3 and Methods). We first examined how the number of detected dots varied across a broad range of fluorescence intensity thresholds and identified 2% as the most suitable threshold for dot detection in this dataset (Extended Data Fig. 10a,b and Methods). We then assessed the effect of Deconwolf on the number of transcripts correctly decoded and on cell type calling, which is based on which transcripts are expressed in each cell. Across the middle temporal gyrus cortex section profiled by ISST, we observed a 3.4-fold increase in the number of transcripts identified in Deconwolf-deconvolved images compared with raw ones (328,437 and 96,934, respectively) (Fig. 6a). The decoded transcripts were distributed along a gradient decreasing from the supragranular to the infragranular extremity of the cortical section, and the transcript counts remained strongly correlated between deconvolved and raw images throughout the length of the section (Fig. 6a and Extended Data Fig. 10c). Only one target gene, *SMYD1*, showed a 50% reduction in transcript counts upon deconvolution of the images, possibly related to the fact that its expression was very low. We then annotated different cell types based on the relative expression of each of the 120 profiled genes in individually segmented cells (Methods). The number of cells being successfully annotated increased from 55% to 75% after applying Deconwolf, as a result of the substantially higher number of cell type-specific genes identified in deconvolved images (Fig. 6b and Extended Data Fig. 10d–f). Altogether, these results demonstrate that Deconwolf can considerably improve the sensitivity of target detection and efficiency of cell type calling in ISST experiments.

### Deconwolf improves the detection efficiency of OligoFISSEQ

Last, we sought to determine whether Deconwolf could also improve the detection sensitivity of OligoFISSEQ^14^, a method that enables reconstruction of DNA trajectories. OligoFISSEQ depends on the colocalization of fluorescent dots generated from the same target locus during multiple rounds of in situ sequencing. Importantly, even though OligoFISSEQ barcodes include redundancies to maximize their detection, the method remains sensitive to the colocalization procedure used to detect the barcodes. We therefore tested the potential of Deconwolf to overcome this limitation by using a previously generated OligoFISSEQ image dataset consisting of 46 DNA loci along the X chromosome that had been visualized together in the same cells using five cycles of in situ sequencing^14^ (Methods). Visual inspection of the images in the original dataset showed densely packed clouds of fluorescent dots in different colors inside each nucleus, which could be only partially resolved by applying the commercial deconvolution software (Nikon NIS Elements AR, v5.02.0) incorporated in the OligoFISSEQ image processing pipeline (Fig. 6c). To test whether Deconwolf would generate more resolved images and improve barcode decoding in OligoFISSEQ, we applied it to the same image dataset, which rendered individual fluorescent dots clearly visible (Fig. 6c). As a result, the efficiency of OligoFISSEQ barcode detection dramatically increased from a mean ± s.d. of 74.1 ± 1.1% to 97.2 ± 0.5% after applying Deconwolf (Fig. 6d). Importantly, when using the NIS deconvolution tool a considerable fraction (48.3%) of the barcodes was consistently detected at relative low frequency (<75%), whereas using Deconwolf 94.4% of the barcodes were detected at high frequency (>90%) (Fig. 6e).

We then applied the same chromosome tracing pipeline that was previously developed to reconstruct chromosome trajectories from OligoFISSEQ data^14^, using the coordinates of the fluorescent dots identified in the Deconwolf images as input. The increase in barcode detection efficiency enabled by Deconwolf yielded multiple complete X chromosome traces for which no interpolation of missing targets was required, whereas using the NIS deconvolution software no complete trace without interpolation could be obtained (Fig. 6f). Such fully decoded traces typically featured more nodes compared with the single-cell traces reconstructed from NIS-deconvolved images (Fig. 6g–j). To quantitatively compare the chromosome traces reconstructed after deconvolution with NIS or Deconwolf, we compared the contact frequency between the 46 DNA loci visualized by OligoFISSEQ with the contact frequency between the same loci assessed by Hi-C^24^. The three-dimensional chromosome traces reconstructed from Deconwolf-deconvolved images had a consistently higher correlation with Hi-C data than the traces reconstructed from images deconvolved with the NIS software, for every distance threshold used to call a pair of DNA loci as being in contact (Fig. 6k). Furthermore, the contact frequency map obtained using NIS-deconvolved images was noisier and had higher contact frequencies near the diagonal, compared with the corresponding map generated from Deconwolf-deconvolved images or compared with Hi-C (Extended Data Fig. 10g,h). Altogether, these results demonstrate that Deconwolf can greatly improve the barcode detection efficiency in OligoFISSEQ experiments and, consequently, the fidelity of chromosome topology reconstructions.

## Discussion

We have developed a user-friendly and open-source deconvolution software, Deconwolf, and shown that it outperforms two of the most commonly used deconvolution tools both in terms of quality and fidelity of the deconvolved images, and in terms of computational speed. The speed-up of Deconwolf will enable researchers to achieve accurate results in realistic times, making our software suitable for deconvolving terabytes of imaging data. Importantly, Deconwolf requires minimal hands-on time given that the user needs to calculate the PSF once for a given set-up and then only specify the number of iterations as a single input parameter for deconvolution. This is achieved by using the PSF Calculator embedded in Deconwolf and specifying only four simple parameters: the objective NA; the refractive index of the immersion oil; the emission maxima of the fluorophore; and the pixel size of the camera. Also, Deconwolf is compatible with any input PSF, and users can supply Deconwolf with an experimentally assessed PSF, whenever available (Supplementary Note 3).

Compared with other deconvolution tools that were recently developed to improve the classic Richardson–Lucy method^4,6,7^, a major advantage of Deconwolf is that it can be readily implemented on an ordinary laptop computer without the need for proprietary software, vendor-specific hardware or tailored training data, and can be used by anyone with a basic knowledge of fluorescence microscopy image analysis and without prior expertise in deconvolution. For example, by running Deconwolf with default settings (that is, specifying only the number of iterations) on an image previously used to showcase the RLN software^7^, we achieved comparable if not superior results (Supplementary Fig. 9) without having to first generate a large image training dataset, which is instead required by RLN given that it leverages deep learning^7^.

Deconwolf managed to solve two major limitations of conventional widefield fluorescence microscopy: the inability to resolve crowded signals; and the need for high-NA objectives to detect (near-)diffraction limited signals. With Deconwolf we managed to reliably count individual transcripts even in highly crowded smFISH images (imaged with high-NA objectives) and tumor tissue sections (with a typical density of signals) imaged with a ×20 air objective. This represents a major advance in the field, enabling the throughput needed in research on clinically relevant material.

Previous attempts to improve the resolvability of crowded signals in smFISH and imaging-based spatial transcriptomics experiments relied on super-resolution or expansion microscopy or a combination of both^25–28^. Here, we have shown that satisfactory results can be achieved using a simple widefield microscope set-up together with Deconwolf. Crucially, although super-resolution and expansion microscopy methods have limited throughput, this is not the case for widefield microscopy combined with Deconwolf. We note, however, that the advantage of Deconwolf on crowded smFISH signals applies to expression levels similar to those that we have shown for *GAPDH*. If the transcript density is higher, expansion microscopy or super-resolution microscopy will most probably outperform widefield imaging coupled with Deconwolf.

A limitation of Deconwolf, as with any other deconvolution tool, is that the accuracy of the deconvolved images will unavoidably be hampered by mismatches between the real PSF of the microscope and the theoretical PSF given as input to the software. Ideally one would image a sample on both a widefield and a super-resolution microscope to fine-tune the PSF model and catch deconvolution artifacts caused by PSF mismatches. However, this is often impractical given time constraints and that super-resolution microscopes might not always be available. One possible solution to reduce deconvolution artifacts due to PSF mismatches would entail using more than one PSF model. Currently, Deconwolf includes only one theoretical PSF model but we anticipate the inclusion of multiple PSF models that the user can choose from. Methods for PSF optimization based on physics principles represent another solution to reduce the negative effect of PFS mismatches, and we will strive to include some of these methods in Deconwolf in the future. In the long run, we also envision that Deconwolf will be equipped with a machine learning module to further enhance the accuracy and performance of the software.

In conclusion, Deconwolf represents a tool that can be adopted across the life sciences to increase the amount of biological information retrieved from a wide range of imaging data. We therefore anticipate that Deconwolf will democratize the use of deconvolution in bioimaging and be used in numerous applications in both research and diagnostics.

## Methods

### Experimental procedure

#### Samples

##### Cell lines

We purchased SKBR3 and hTERT RPE-1 cells from American Type Culture Collection (cat. no. HTB-30 and CRL-4000, respectively), U-2 OS cells from CLS Cell Lines Service (cat. no. 300174), and HAP1 cells from Horizon Discovery (cat. no. C859). We cultured SKBR3 cells in McCoy’s 5A (Sigma-Aldrich, cat. no. M9309) supplemented with 10% heat-inactivated fetal bovine serum (Sigma-Aldrich, cat. no. F9665); U-2 OS cells in DMEM, high glucose, GlutaMAX Supplement, pyruvate (Thermo Fisher Scientific, cat. no. 10569010), supplemented with 10% (vol./vol.) fetal bovine serum (Sigma-Aldrich, cat. no. F9665); and HAP1 cells in Iscove’s Modified Dulbecco’s Medium (Sigma-Aldrich, cat. no. I2911) supplemented with 10% fetal bovine serum (Sigma-Aldrich, cat. no. F9665). We incubated the cells at 37 °C in 5% CO_2_ and 5% O_2_ (for RPE-1 cells only). All three cell lines were tested for *Mycoplasma* contamination and were negative. We did not authenticate any of the cell lines used. None of the cell lines used is included in the International Cell Line Authentication Committee database of commonly misidentified cell lines.

##### Human brain tissue microarray

For GFAP immunofluorescence in human cerebral cortex tissue sections, we used a tissue microarray (TMA) previously constructed by Atlas Antibodies from formalin-fixed paraffin-embedded tissue samples that were purchased from the BioIVT biobank. This TMA can be used for biomarker and assay validation without the need for an ethical approval.

##### Tumor tissue microarray

For smFISH at low (×20) magnification, we purchased multiple 5-μm-thick frozen tissue sections cut from a TMA containing 28 cores (including 14 different tumor samples and 14 normal tissues) from US Biomax (cat. no. FMC282e). This TMA can be used for biomarker and assay validation without the need for an ethical approval.

#### smFISH

##### smFISH in SKBR3 cells

We designed and produced an smFISH probe targeting different isoforms of the *GAPDH* gene (Supplementary Table 1) using the iFISH pipeline that we previously developed to produce oligonucleotide-based DNA FISH probes^13^. We performed hybridization for 16–18 h at 30 °C in a humidity chamber using RNA hybridization buffer containing 25% formamide (Millipore, cat. no. S4117), 2X SSC, 10% dextran sulfate (Sigma-Aldrich, cat. no. D8906-50G), 1 mg ml^−1^ *Escherichia coli* transfer RNA (Sigma-Aldrich, cat. no. R1753-2KU), 0.02% bovine serum albumin (Thermo Fisher Scientific, cat. no. AM2616) and 10 mM vanadyl-ribonucleoside complex (New England Biolabs, cat. no. S1402S). After hybridization, we washed the cells in RNA wash buffer containing 25% formamide and 2X SSC at 30 °C for 30 min. We then hybridized secondary fluorescently labeled oligonucleotides at a final concentration of 20 nM in RNA hybridization buffer for 3 h at 30 °C in a humidity chamber, followed by one wash with RNA wash buffer at 30 °C, and 30 min incubation at 30 °C in 2X SSC, 25% formamide and 1.23 ng ml^−1^ Hoechst 33342 (Thermo Fisher Scientific, cat. no. 62249). Before imaging, we mounted the samples with 2X SSC, 0.4% glucose (Sigma-Aldrich, cat. no. G8270), 10 mM Tris-HCl (Merck, cat. no. 1185-53-1), 10 mM Trolox (Sigma-Aldrich, cat. no. 238813), 37 ng μl^−1^ glucose oxidase (Sigma-Aldrich, cat. no. G2133) and 32 mM catalase (Sigma-Aldrich, cat. no. C3515). To image the samples we used a custom-built Eclipse Ti-E inverted widefield microscope system (Nikon) controlled by the NIS Elements software (Nikon) and equipped with a ZYLA 4.2P sCMOS camera (Andor Technology) using the CFI Plan Apochromat Lambda 1.45 NA ×100 oil Nikon objective. For each sample, we acquired image stacks spanning 8–15 μm with 0.2–0.6 μm stepwise between consecutive focal planes.

##### smFISH on tissue microarray

We designed and fluorescently labeled a classical smFISH probe (in which each oligonucleotide in the probe is directly conjugated to one fluorophore) targeting different isoforms of the *MKI67* gene using the pre-designed oligonucleotide database that we previously created^30^. The genomic coordinates and corresponding oligonucleotide sequences are available in Supplementary Table 1. We performed smFISH on frozen tissue sections from the tumor TMA described above, following a procedure adapted from ref. ^31^. In brief, we fixed the sections with 1× PBS (Thermo Fisher Scientific, cat. no. AM9625) and 4% paraformaldehyde (Thermo Fisher Scientific, cat. no. 11481745) at room temperature, and then rinsed the sections twice with 1x PBS at room temperature, followed by two rinses with ice cold 70% ethanol and then incubated the sections in the same solution for 3 h at 4 °C. We then rehydrated the sections by replacing the 70% ethanol with the RNA wash buffer described above. We performed all steps from hybridization until imaging as described above for SKBR3 cells. We imaged the sample on a Nikon Eclipse Ti-E widefield microscope, using either the CFI Plan Apochromat Lambda 1.4 NA ×60 oil or the CFI Plan Apo VC 0.75 NA ×20 air objective (Nikon).

### iFISH

We designed and produced iFISH probes targeting 63 loci all along human chromosome 16 (Extended Data Fig. 7a) using the iFISH pipeline that we previously developed^13^. We cultured HAP1 cells on 22 × 22 mm coverslips (VWR, Nr. 1.5, cat. no. 631-0125) in 6-well plates (Merck, cat. no. CLS3506). Once the cells reached around 90% confluency on each coverslip, we fixed them with 1× PBS (Thermo Fisher Scientific, cat. no. AM9625) and 4% paraformaldehyde (EMS, cat. no. 15710) for 10 min at room temperature, followed by quenching of unreacted paraformaldehyde in 1× PBS and 125 mM glycine (Fisher Scientific, cat. no. 10467963) for 5 min at room temperature. Subsequently, we washed the cells three times, 5 min each with 1× PBS (Thermo Fisher Scientific, cat. no. AM9625) and 0.05% Triton X-100 (Promega, cat. no. H5142) at room temperature, and permeabilized them in 1× PBS and 0.5% Triton X-100 for 20 min at room temperature, followed by three more washes with 1× PBS and 0.05% Triton X-100 at room temperature, for 5 min each. We then incubated the cells in 0.1 N HCl for 5 min at room temperature, followed by two washes in 1× PBS and 0.05% Triton X-100 at room temperature, for 5 min each, and rinsing with 2× SSC buffer (Thermo Fisher Scientific, cat. no. AM9763). We stored the cells in 2× SSC supplemented with 0.05% NaN_3_ (Merck, cat. no. S2002) at +4 °C for up to 1 month until hybridization. Prior to hybridization, we incubated the cells in 50% formamide, 2× SSC and 50 mM phosphate buffer overnight at room temperature, followed by 1 h of incubation with a pre-hybridization buffer containing 50% formamide, 2× SSC, 5× Denhardt’s solution (Thermo Fisher Scientific, cat. no. 750018), 50 mM sodium phosphate buffer (home made from sodium dihydrogen phosphate (Merck, cat. no. 7558-80-7) and disodium hydrogen phosphate (Merck, cat. no. 7558-79-4)), 1 mM EDTA (Thermo Fisher Scientific, cat. no. AM9260G), 100 mg ml^−1^ salmon sperm DNA (Thermo Fisher Scientific, cat. no. 15632011) and 100 mg ml^−1^ human Cot-1 DNA (Thermo Fisher Scientific, cat. no. 15279011), pH 7.5–8 at 37 °C in a humidity chamber. Meanwhile, we prepared a hybridization mix containing the probes of interest diluted tenfold with 1.1× hybridization buffer containing 55% formamide, 2.2× SSC, 5.5× Denhardt’s solution, 55 mM sodium phosphate buffer, 1.1 mM EDTA, 100 μg ml^−1^ salmon sperm DNA, 100 μg ml^−1^ human Cot-1 DNA and 11% dextran sulfate, pH 7.5–8. The final concentration of each probe in the hybridization mix is 0.06 nM per DNA oligonucleotide. We designed and produced all of the probes using the iFISH pipeline, which we previously developed^13^. The genomic coordinates and corresponding oligonucleotide sequences of all of the probes are listed in Supplementary Table 2. After pre-hybridization, we removed the pre-hybridization buffer and replaced it with the hybridization mix, sealing the coverslips with Fixogum (Triolab, cat. no. LK071A) to prevent any leakage. We denatured the samples at 75 °C for exactly 1 min and 10 s, followed by incubation at 37 °C overnight in a sealed humidity chamber. The next day, we rinsed the coverslips with 2× SSC and 0.02% Tween, followed by two washes, 5 min each, with 0.2× SSC and 0.2% Tween (Sigma-Aldrich, cat. no. P9416) pre-warmed at 60 °C, a quick rinse with 4× SSC and 0.2% Tween and then 2× SSC, and a final wash with 2× SSC and 25% formamide. We then prepared a second hybridization mix containing the secondary fluorescently labeled oligonucleotides at a final concentration of 20 nM per oligonucleotide in 2× SSC, 25% formamide, 10% dextran sulfate, 1 mg ml^−1^ *E.* *coli* tRNA (Sigma-Aldrich, cat. no. R1753-2KU) and 0.02% bovine serum albumin (Sigma-Aldrich, cat. no. A9418). We incubated the samples at 30 °C overnight in a sealed humidity chamber. The next day, we washed the coverslips with 2× SSC and 25% formamide at 30 °C for 1 h, followed by 30 min incubation with 1 ng ml^−1^ Hoechst 33342 in 2× SSC and 25% formamide at 30 °C, and two washes with 2× SSC, 5 min each. We mounted and imaged the samples on a Nikon Eclipse Ti-E widefield microscope equipped with an iXON Ultra 888 EMCCD camera (Andor Technology) using the CFI Plan Apochromat Lambda 1.45 NA ×100 oil objective (Nikon).

#### Immunofluorescence

##### GFAP immunofluorescence on human brain tissue

We cut a 4-μm-thick tissue section from the human brain TMA described above and baked it at 55 °C on a heating plate for 25 min. Afterwards, we manually dewaxed the section and performed heat-induced epitope retrieval (HIER) in a pH 6 citrate buffer (Sigma-Aldrich, cat. no. C9999) in a pressure cooker (Bio SB TintoRetriever) at 114–121 °C for 20 min. We incubated the section with an anti-GFAP primary antibody (Atlas Antibodies, cat. no. AMAb91033) diluted 1:500 (vol/vol) in TNB buffer containing 0.1 M Tris-HCl, 0.15 M NaCl and 0.5% blocking reagent (Akoya, cat. no. SKU FP1020), pH 7.5, and incubated the section overnight at 4 °C. The next day, we incubated the section with a goat anti-mouse secondary antibody coupled with AlexaFluor 555 (Thermo Fisher Scientific, cat. no. A-21424) diluted 1:800 (vol/vol) in TNB buffer supplemented with 4′,6-diamidino-2-phenylindole (DAPI, Thermo Fisher Scientific, cat. no. D1306) for 90 min at room temperature. Last, we mounted the section with Fluoromount-G (Thermo Fisher Scientific, cat. no. 00-4958-02) and imaged it either with a Nikon Eclipse Ti-E widefield microscope equipped with a ZYLA 4.2P sCMOS camera (Andor Technology) and using a CFI Plan Apochromat Lambda 1.4 NA ×60 oil objective (Nikon), or with a Leica SP8 confocal microscope (DMi8-CS) equipped with a ×63 HC PL APO 1.40 oil CS2 objective (Leica Microsystems). For the widefield imaging we used the following settings: 16 bit acquisition; Z-step size, 0.25 μm; number of steps, 41; while for the confocal imaging we used the following settings: 16 bit acquisition; zoom factor, 1.4; pinhole, 1 Airy unit; line average, 4; Z-step size, 0.25 μm; number of steps, 61. For the confocal imaging we adjusted the detector gain measuring the signal of the antibody to 10 V. To compare widefield and confocal images, we imaged the same region of interest consisting of 4 × 4 tiles with 10% overlap.

##### Tubulin immunofluorescence on U-2 OS cells

We seeded the cells on coverslips (Ø 18 mm, 1.5 Marienfeld high precision, VWR International, cat. no. 630-2200) and kept them growing for 1–3 days at 37 °C in 5% CO_2_. We fixed the cells with a pre-warmed solution of 8% formaldehyde (Electron Microscopy Sciences, cat. no. 15710) in 1× PBS for 10 min at room temperature. We permeabilized the cells with 0.5% (vol./vol.) Triton X-100 in 1× PBS and blocked with 5% (w/vol) BSA in 1× PBS and 0.1 M glycine for 30 min at room temperature. We stained the cells using a primary antibody against alpha-tubulin (Sigma-Aldrich, cat. no. T6074) diluted 1:250 in 5% BSA (w/vol) in 1× PBS and 0.1 M glycine for 1 h at room temperature. After five washing steps with 1× PBS and blocking with 5% BSA in 1× PBS and 0.1 M glycine, we incubated the samples with a secondary antibody (Goat Anti-Mouse AlexaFluor 555, Abcam, cat. no. ab150118, 1:400 dilution) and DAPI (Thermo Fisher Scientific, cat. no. D1306) for 1 h at room temperature. Last, we washed the samples five times with 1× PBS and mounted them in Prolong Gold (Thermo Fisher Scientific, cat. no. P10144). We imaged the cells with either a Nikon Eclipse Ti-E widefield microscope equipped with an iXon Ultra 888 EMCCD camera (Andor Technology) and using a Plan Apochromat Lambda 1.45 NA ×100 oil objective (Nikon) or with an LSM 980 confocal microscope (Carl Zeiss) equipped with a Plan Apochromat 1.4 NA ×63 oil objective (Carl Zeiss). For the widefield imaging we used the following settings: 16 bit acquisition; x–y pixel size, 129.8 nm; z-step size, 0.25 μm; number of steps, 81. For the confocal imaging we used the following settings: 16 bit acquisition; zoom factor, 5.0 (x–y pixel size, 26.3 nm); pinhole, 1 Airy unit; line average, 2; scanning speed, 10 (pixel dwell time, 0.42 μs); z-step size: 0.3 μm; number of steps: 52. To compare the widefield and the confocal images, we imaged the same field of view, starting with confocal acquisition.

### Confocal and STED imaging of nuclear pores

We visualized nuclear pores in human PtK2 cells using immunofluorescence with a primary antibody against the nuclear pore protein Nup153 (Abcam, cat. no. ab24700) diluted to 1 μg ml^−1^ and a goat anti-mouse IgG secondary antibody (Merck, cat. no. ST635P-1002-500UG, 1:400 dilution). We imaged the samples using confocal and STED super-resolution microscopy on a Leica SP8 3X STED system at the Advanced Light Microscopy facility at SciLifeLab. We used a Leica pulsed white-light laser (selectable excitation wavelengths, 470–670 nm) and an excitation wavelength of 490 nm to excite Oregon Green 488 fluorophores on immunolabeled nuclear pore complexes, and a continuous-wave laser (592 nm, MPB Communications) for depletion. For imaging, we used a chromatically optimized oil immersion objective (HC PL APO ×100/1.40 OIL STED WHITE, Leica Microsystems). We passed fluorescence signals through a 0.9 Airy unit pinhole, a dichroic mirror optimized for fluorescence detection, and a notch STED filter placed in front of a sensitive photodetector (Leica Hybrid Detectors). We scanned frames (1,024 × 1,024 pixels) at a speed of 200 lines per second with 4-line averages using a pixel size of 25 nm for STED and thereafter confocally at 1,000 lines per second.

### Computational procedure

#### Deconwolf

Deconwolf is based on the Richardson–Lucy method^2,3^ with the SHB^15^ acceleration technique together with a positivity constraint. Furthermore, Deconwolf implements a 3D image boundary handling approach based on a 2D method originally developed in astrophysics^21^, which extends the image and treats what is outside of it as missing information. This approach makes it possible to process large images in tiles, with minimal artifacts. Deconwolf is written in the C programming language, it uses the FFTW3 package^17^ for fast Fourier transforms, and utilizes savings in memory and speed, given that the images are real and not complex. All internal computations are done using 32 bit floating point precision regardless of the input and output formats. Deconwolf can read and write TIFF stacks of either 16 bit unsigned integers or 32 bit floating point data. FFTW3 is already fully parallelized and further parallelization is enabled using POSIX Threads and the OpenMP library. Deconwolf can be run as a command line interface or accessed from a graphical user interface (https://github.com/elgw/deconwolf-gui) written in C using GTK. The entire Deconwolf package, including extensive usage documentation, is available at https://github.com/elgw/deconwolf/. Below, we describe some of the key features of Deconwolf in detail.

##### Point spread function calculator

There are a multitude of PSF models available for widefield microscopy (reviewed in ref. ^32^ and discussed in Supplementary Note 3). For convenience, Deconwolf is equipped with a PSF calculator (PC) based on the Born–Wolf model^19^. The derivation of the Born–Wolf model is based on several assumptions, including ideal imaging conditions, which in practice can never be achieved. Nevertheless, we have found this model to perform very well on all of the images that we have tested thus far. In Deconwolf, we rasterize the PSF by integrating over the lateral extent of each pixel, that is, by assuming that each sensor pixel is a perfect square, and that no additional low-pass filtering is present in the system. In contrast, the PSF Generator tool^20^ samples the PSF only at the center of each pixel, which can be problematic because, under the Born–Wolf model, the PSF (at normal pixel sizes) cannot be sampled densely enough according to the Nyquist–Shannon sampling theorem. To achieve high performance, Deconwolf pre-computes the Born–Wolf integral for a discrete set of radii (*r*) at all relevant depths (*z*), inspired by PSF Generator. Then, using radial symmetry, the Born–Wolf integral can be interpolated for any (*x, y, z*) from the pre-computed values. While PSF Generator uses linear interpolation, Deconwolf interpolates the radial profile using Lanczos-3 interpolation assuming symmetry around *r* = 0.

##### Processing large images with Deconwolf (automatic tiling)

Deconwolf can process large images, such as whole-slide images, even on a standard laptop computer, given that the software is able to read and/or write a small portion of the images on demand. Specifically, Deconwolf has an option to process the data in tiles over the lateral plane (the full axial size is always used). This process is completely transparent to the user, who must specify only the largest allowed tile size that Deconwolf should use. Optionally, the user can tune a parameter that controls the quality of this procedure, that is, how much the tiles should overlap. The tile processing consists of the following internal steps:

1. The input tif image is streamed to the disk as raw float data, never loading the whole image into RAM.
2. A tiling grid is set up to divide the lateral domain of the image into the specified number of tiles, *T*. The grid size is at most *T* × *T* × *P* pixels, where *P* is the axial size of the image.
3. One tile at a time is streamed from the disk, including extra overlap or padding *p* (except at the image edge) from neighboring tiles. The tile is deconvolved and streamed back to the disk.
4. For the pixels in regions where the tiles overlap due to padding, the values are weighed linearly by the distance from the tile edges.
5. The raw output image is converted to a tif file without loading the full image into RAM.

For up-to-date descriptions of how to use the tiling option, please see the tutorials and documentation included in the Deconwolf package available at https://github.com/elgw/deconwolf/.

### Generation of in silico smFISH images

To generate ground truth synthetic smFISH images, we started with volumetric images of size 255 × 255 × 40 pixels, where each pixel is a square of 130 × 130 × 130 nm. We generated diffraction limited dots by placing Gaussian blobs with a total intensity of 10,000 and a sigma equal to 0.7 pixels at random locations with sub-pixel accuracy (that is, we integrated the Gaussians over each pixel). We then added a constant intensity background equal to 1,000. To emulate a real smFISH image, we convolved the ground truth image with the PSF generated by Deconwolf based on the Born–Wolf model and then added Poissonian noise. Last, we added Gaussian noise with a sigma of 10 to simulate sensor noise. Note that we used the same PSF to generate the synthetic images (convolution) and to deconvolve them, which is an ideal scenario. However, in the case of real images, there will always be a mismatch between the true PSF of the microscope and the PSF used for deconvolution. Hence the results of the analyses performed on synthetic images must be interpreted as upper bounds or best-case performance.

### Dot detection in smFISH and iFISH images

To detect fluorescence dots in smFISH and iFISH images, we used our in-house analysis suite DOTTER (v.0.598, available at github.com/elgw/dotter) written in MATLAB (R2020a) and C99 with GSL (https://www.gnu.org/software/gsl/), which is specifically designed to detect (near-)diffraction limited dots in smFISH and iFISH images. In brief, we detected all local maxima in the images using 6-connectivity, that is, we considered a pixel as a local maximum only if it was brighter than its face neighbors. We then ranked the identified dots based either on their brightness (intensity-based dot detection) or on their value after filtering the image with a difference of Gaussians (DoG-based dot detection).

### Comparison of iFISH dots before and after Deconwolf-deconvolution

To compare the number and features of the dots identified by DOTTER in iFISH images before (raw) and after deconvolution with Deconwolf, we used a custom script written in MATLAB (R2020a), which, for every dot in a raw image, searches for the nearest dot in the corresponding deconvolved image and vice versa. In brief, we looped through each segmented nucleus in the raw and deconvolved images and extracted the 3D coordinates of each dot, separately for each of the four fluorescence channels corresponding to different subsets of 63 iFISH probes targeting 63 DNA loci in different A and B subcompartments along chromosome 16 (Extended Data Fig. 7a). We excluded nuclei in which no dots were found in the raw or in the deconvolved or in both images. For each nucleus and channel, we used the knnsearch function in MATLAB to find, for every dot detected in a raw image, the nearest neighbor dot in the corresponding deconvolved image (labeled as ‘Raw&DW’) and vice versa (‘DW&Raw’), using a threshold of 260 nm (that is, two dots closer than 260 nm were considered as matching between raw and deconvolved images). We labeled the dots identified in the raw images but not in the corresponding deconvolved images, as ‘Lost’, whereas we labeled as ‘New’ the dots identified in the deconvolved images but not in the corresponding raw ones. For each dot group we then plotted the distributions of the FWHM value and of the nuclear–contrast ratio of the dots, defined as the ratio of the fluorescence intensity of the pixels corresponding to the local maxima to the average fluorescence intensity of the corresponding nuclei (Fig. 4m,n and Supplementary Fig. 8d–i).

### Quantification of smFISH dots in ×20 magnification images

We imaged 10 field of view (FOVs) of a breast adenocarcinoma tissue core in a TMA on which we performed smFISH with a probe targeting *MKI67* gene transcripts (Experimental Procedure), using a widefield inverted epifluorescence microscope (Nikon Ti-E) with a ×20 air objective (Nikon CFI Plan Apo VC ×20). For a subset of the regions imaged with the ×20 objective, we also acquired images using a ×60 oil objective (Nikon CFI Plan Apochromat Lambda 60XC). We detected dots in all of the images at both magnifications using the DoG-based dot detection module in DOTTER (see above). We generated 2D nuclear segmentation masks for each FOV at ×60 magnification (NA = 1.4) using a random forest classifier that we previously trained on pixel features (available at https://github.com/elgw/pixelClassifier) and an ad hoc Python script available at https://github.com/ggirelli/deconwolf-tissue-smFISH. We assigned pixels within the intersection of nuclear masks to the nucleus with the closest border in the original mask. We then rescaled the masks to a resolution matching the corresponding FOV imaged at ×20 magnification using the skimage.transform.rescale function in Python. We discarded any object with a size below 1,500 pixels from the mask and assigned the smFISH dots detected by DOTTER to each mask based on their 2D coordinates. We selected dots with an FWHM between half a pixel side and 5 pixel sides and rescaled the intensity of the dots based on a rescaling factor reported in the corresponding Deconwolf log file. To distinguish between bona fide transcripts and putative false-positive dots, we filtered the dots based on a threshold intensity corresponding to a visual local minimum in the log-transformed DoG-filtered intensity distribution of all of the dots in each FOV. To automatically identify a threshold for distinguishing true dots from noise, we defined a metric, which we named contrast-to-noise ratio (CNR), as follows:

1 $${{\mathrm{CNR}}}=\frac{{{\mathrm{signal}}}{{\mbox{-}}}{{\mathrm{background}}}}{{{\mathrm{noise}}}}$$

where Signal is the pixel value of the dot (identified as a local maximum). To calculate Background and Noise, we sampled an image at a distance of 5 pixels from each dot (using linear interpolation in 100 points) and defined Background and Noise as the mean and standard deviation of the samples, respectively. To compare the locations and counts of the dots identified in the same FOV imaged at ×60 or ×20, we first applied an ad hoc MATLAB script (available at https://github.com/ggirelli/deconwolf-tissue-smFISH) to find the best translation between the corresponding images and to minimize the local pairwise distances by allowing deformations according to a second-order polynomial. We then quantified the percentage of dots identified at ×60 magnification (NA = 1.4) (for both the raw and the deconvolved images) that were also detected in the corresponding ×20 FOVs, and vice versa.

### ISST image analysis and cell typing

We retrieved z-stack images from an ISST dataset, which we previously generated to visualize 120 different gene transcripts in a tissue section from the human middle temporal gyrus of a 38-year-old male donor affected by epilepsy^23^. ISST images were acquired using ZEN (v2.3) microscopy software (Zeiss). To decode individual transcripts in images before (raw) or after deconvolution with Deconwolf, we used an in-house pipeline (available at https://github.com/Moldia/iss_starfish/), which incorporates the Starfish package^33^. In brief, we first aligned images of the same FOV obtained at each hybridization-imaging cycle and stitched multiple FOVs together using the Microscopy Image Stitching Tool (MIST)^34^. We then used the FindSpots module in Starfish with a masking radius of 15 to localize individual transcripts. We changed the thresholding parameter (which specifies the absolute lower bound for scale space maxima) to compare deconvolved and raw images. Finally, we used probabilistic cell typing^35^ to assign cells to one of 18 brain cell types previously described^29^ based on the type and abundance of transcripts detected in each segmented cell.

### OligoFISSEQ image analysis and chromatin tracing

We retrieved raw images from the ChrX-46plex O-eLIT dataset that we previously generated by OligoFISSEQ^14^ and deconvolved using a proprietary software (Nikon NIS Elements AR v5.02.01) implementing the Richardson–Lucy method^2,3^. To compare images deconvolved with NIS or Deconwolf, we manually matched *n* *=* 168 segmented nuclei between corresponding images and applied the two-tier, every-pixel automated OligoFISSEQ analysis pipeline^14^ to identify true signals representing the targeted DNA loci. To reconstruct individual chromosome topologies, we used the Chromosome tracing pipeline as previously used in ref. ^14^. Using this approach, we reconstructed 196 and 172 individual chromosome topologies in Deconwolf- and NIS-deconvolved images, respectively.

### Reporting summary

Further information on research design is available in the Nature Portfolio Reporting Summary linked to this article.

## Online content

Any methods, additional references, Nature Portfolio reporting summaries, source data, extended data, supplementary information, acknowledgements, peer review information; details of author contributions and competing interests; and statements of data and code availability are available at 10.1038/s41592-024-02294-7.

### Supplementary information

Supplementary Information Supplementary Figs. 1–9, Supplementary Notes 1–4, Supplementary References

Reporting Summary

Supplementary Table **Supplementary Table 1**. List of oligonucleotides composing the smFISH probes targeting *GAPDH* and *MKI67* gene transcripts. **Supplementary Table 2**. List of oligonucleotides composing the iFISH probes targeting 65 loci along chromosome 16. **Supplementary Table 3**. List of human genes previously visualized using ISST in human middle temporal gyrus cortex.

### Source data

Source Data Figs. 1d,l, 2k, 4e,l–n, 6d–f,k, Extended Data Figs. 1a,b,d,g, 4e, 6c,h and Supplementary Data Tabulated data to reproduce the plots in Fig. 1d,l, Tabulated data to reproduce the plot in Fig. 2k. Tabulated data to reproduce the plots in Fig. 4e,l–n. Tabulated data to reproduce the plots in Fig. 6d–f,k. Tabulated data to reproduce the plots in Extended Data Fig. 1a,b,d,g. Tabulated data to reproduce the plot in Extended Data Fig. 4e. Tabulated data to reproduce the plots in Extended Data Fig. 6c,h. Tabulated data to reproduce the plots in Supplementary Fig. 8.

## Data Availability

The images of synthetic microtubules, fluorescent rods and *C.* *elegans* whole embryo used for Deconwolf benchmarking can be downloaded from http://bigwww.epfl.ch/deconvolution/index.html - data. Source data to reproduce all of the figures, including raw and deconvolved images, imaging and deconvolution settings, and tabulated data to reproduce all of the plots are available on Figshare at: https://figshare.com/s/64d00b42a5d0c5178c19. Tabulated data to reproduce smaller plots in the main Figures and Extended Data Figures are also available as a separate Source Data file. The RLN image shown in Supplementary Fig. 9a was downloaded from 10.1038/s41592-022-01652-7 (ref. ^7^) (see Fig. 5 in ref. ^7^). iFISH probes were designed using the human genome assembly GRCh38/hg38. smFISH probes were designed using the ENSEMBL transcript IDs listed in Supplementary Table 1. Source data are provided with this paper.

## Extended data

are available for this paper at 10.1038/s41592-024-02294-7.

## Code availability

The entire Deconwolf package can be freely downloaded from https://deconwolf.fht.org/ and is also available at https://github.com/elgw/deconwolf/.
