# Supplementary material for: Deconwolf enables high-performance deconvolution of widefield fluorescence microscopy images
Source: Nat Methods. 2024 Jun 6;21(7):1245–56. doi: 10.1038/s41592-024-02294-7 (PMC11239506; doi:10.1038/s41592-024-02294-7)
Supplement: Supplementary file 1 — Supplementary Figs. 1–9, Supplementary Notes 1–4, Supplementary References [file 41592_2024_2294_MOESM1_ESM.pdf]

nature methods

Supplementary information https://doi.org/10.1038/s41592-024-02294-7

# Deconwolf enables high-performance deconvolution of widefield fluorescence microscopy images

In the format provided by the authors and unedited

## SUPPLEMENTARY INFORMATION

# Table of content


1. Supplementary Figures pg. 2
2. Supplementary Notes pg. 14
3. Supplementary References pg. 21

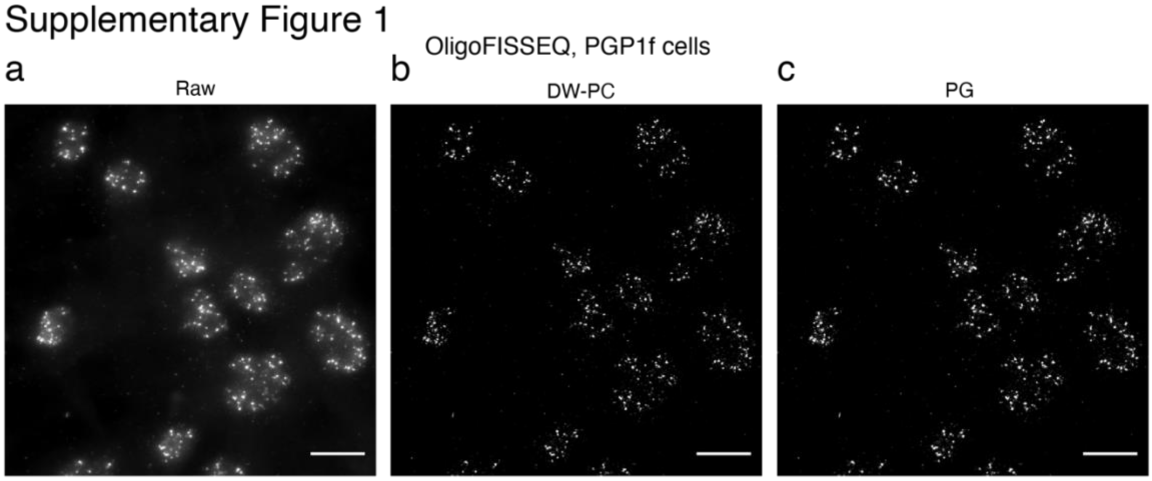


**Supplementary Fig. 1.** Deconwolf is equipped with a high-precision point spread function (PSF) calculator (PC), which yields sharper images after deconvolution compared to the commonly used PSF Generator (PG)[1]. (**a-c**) Maximum z-projection of a z-stack image from the previously published ChrX-36plex OligoFISSEQ dataset[2] without deconvolution (Raw) (a) and after deconvolution with DW either using a PSF generated with the PC tool implemented in DW (b) or with PG (c). Scale bars, 20 µm. All micrographs are from a single experiment.

Supplementary Figure 2

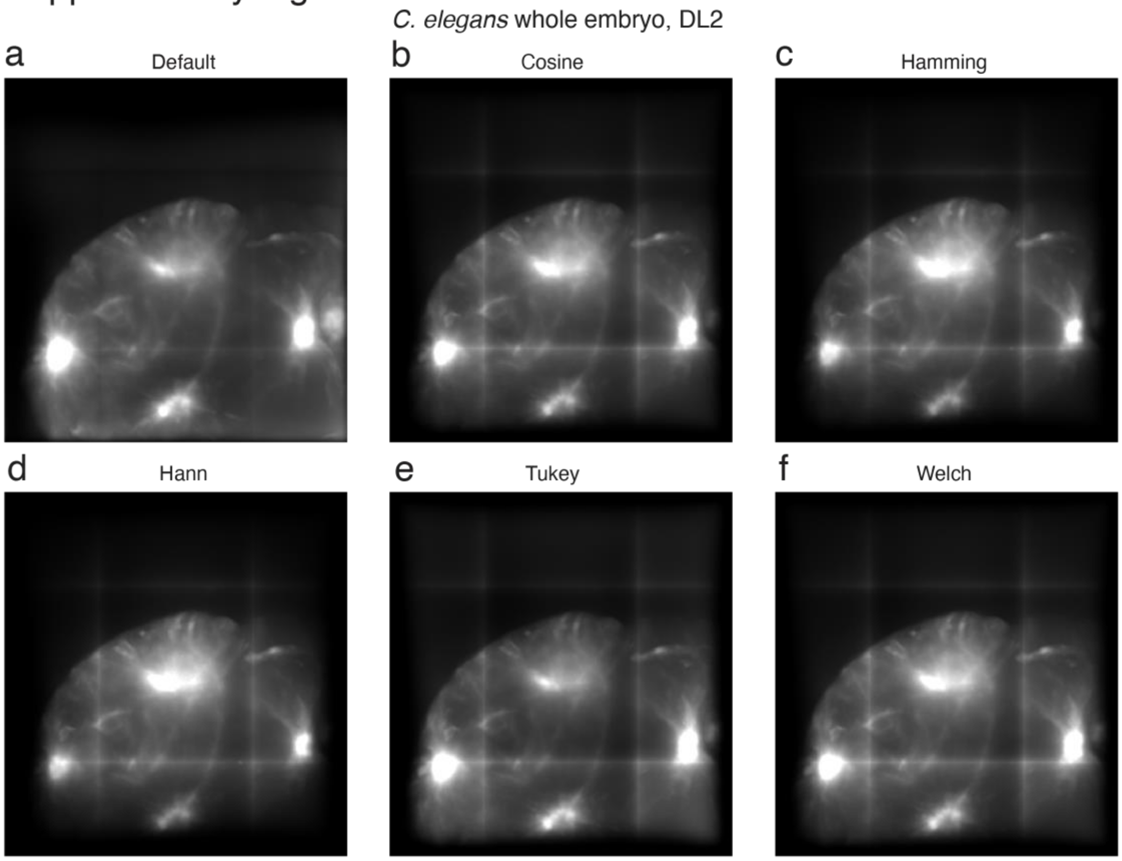


**Supplementary Fig. 2.** Boundary handling in DeconvolutionLab2 (DL2). (**a-f**) Upper left tile of the *C. elegans* whole-embryo image shown in **Fig. 2b** after deconvolution with DL2 using no boundary handling option (Default) or one of the apodization options (plot titles) available in DL2.

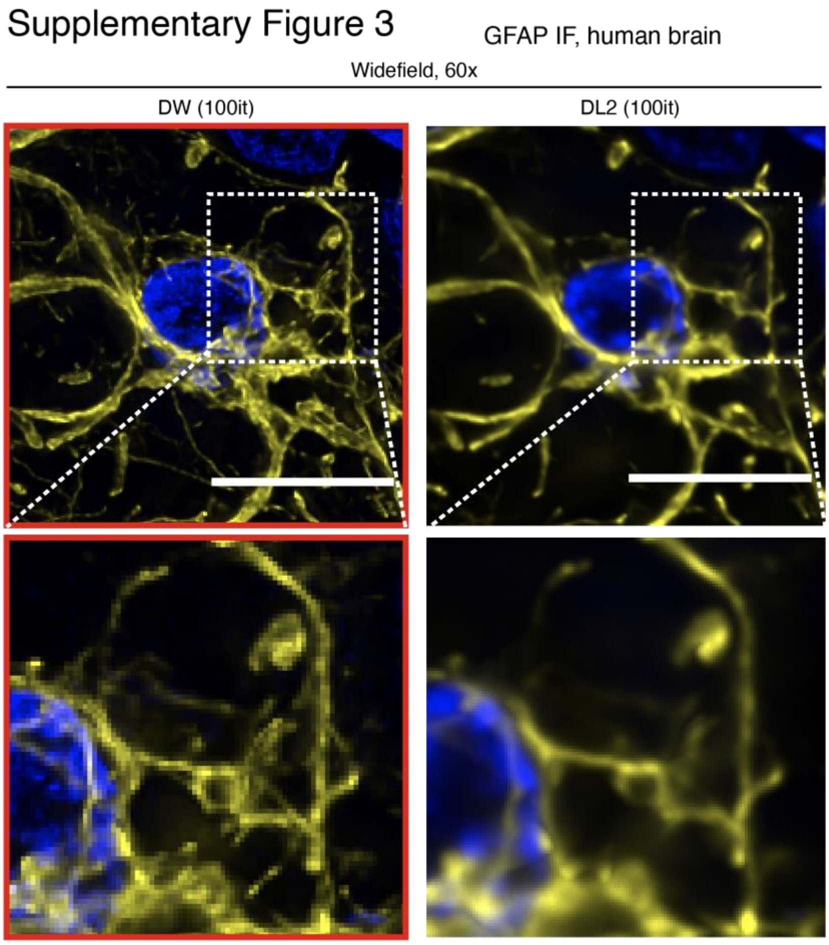


**Supplementary Fig. 3.** Deconwolf (DW) outperforms DeconvolutionLab2 (DL2) on standard immunofluorescence images acquired with a widefield microscope. Maximum z-projection of a z-stack image of a nucleus from a human brain tissue section stained with an antibody against the Glial Fibrillary Acidic Protein (GFAP) (yellow) and imaged on a widefield microscope using a 100x (NA=1.45) oil objective, comparing DW with DL2. Blue, DNA. Scale bars, 10 μm. All micrographs are from a single experiment.

Supplementary Figure 4

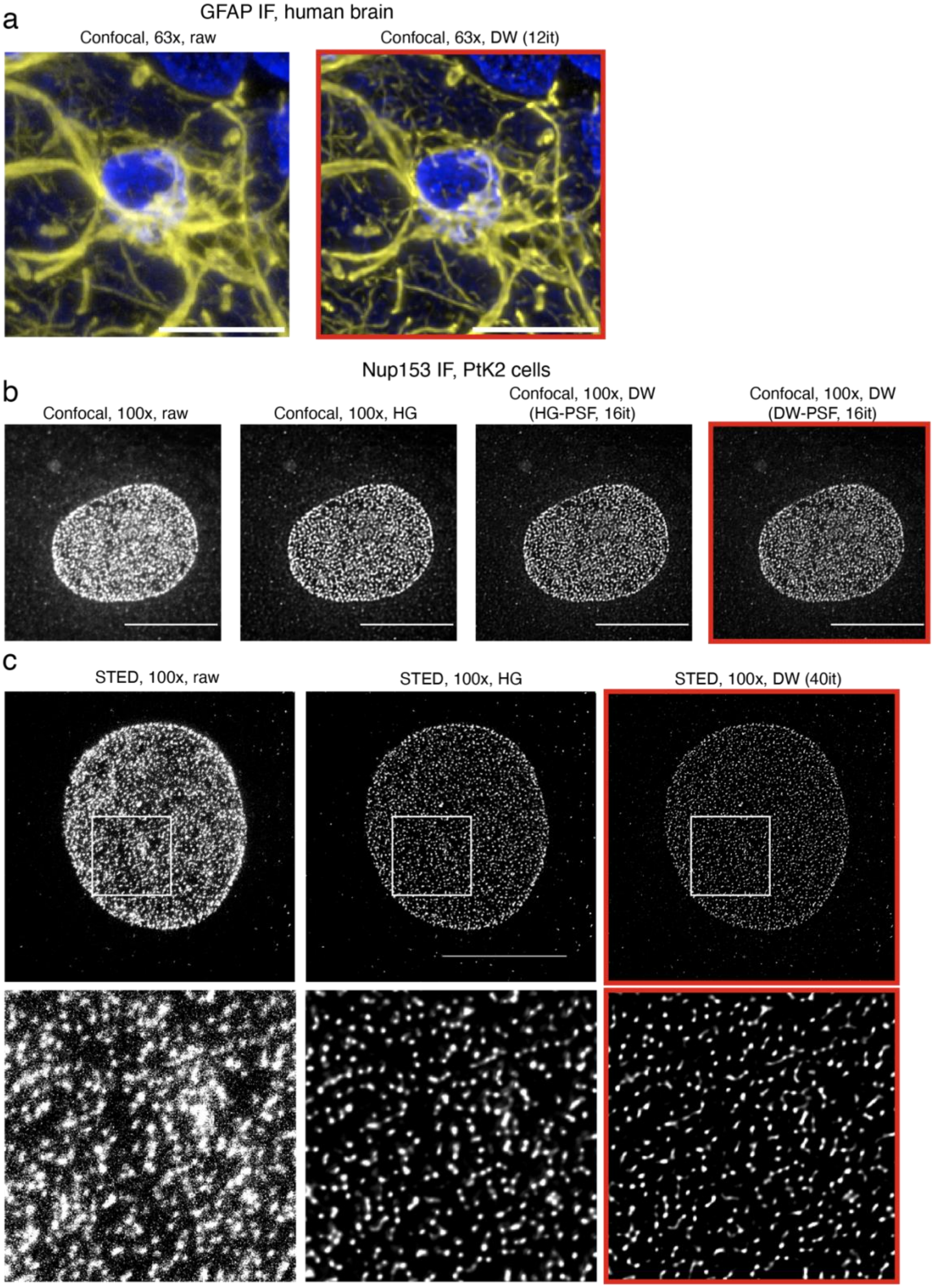


**Supplementary Fig. 4.** Deconwolf (DW) enhances the quality of confocal as well as super-resolution microscopy images. (**a**) Maximum z-projection of a z-stack image of a nucleus from

a human brain tissue section stained with an antibody against the Glial Fibrillary Acidic Protein (GFAP) (yellow) and imaged on a confocal microscope with a 63x oil objective, with or without deconvolution with DW. Blue, DNA. Scale bars, 10 μm. (**b**) Maximum z-projection of a z-stack image of a cell nucleus from human PtK2 cells stained with an antibody against the nuclear pore Nup153 protein and imaged using a confocal microscope of the Advanced Light Microscopy facility at SciLifeLab with or without deconvolution by Huygens (HG) or DW using a point spread function (PSF) calculated either by HG or using the Born-Wolf (BW) model[3] implemented in DW. (**c**) As in (b) but showing an image acquired using a stimulated emission depletion (STED) microscope at the Advanced Light Microscopy facility at SciLifeLab with or without deconvolution by HG or DW. All scale bars are 10 μm. DW-deconvolved images are framed in red. All micrographs shown are from a single experiment.

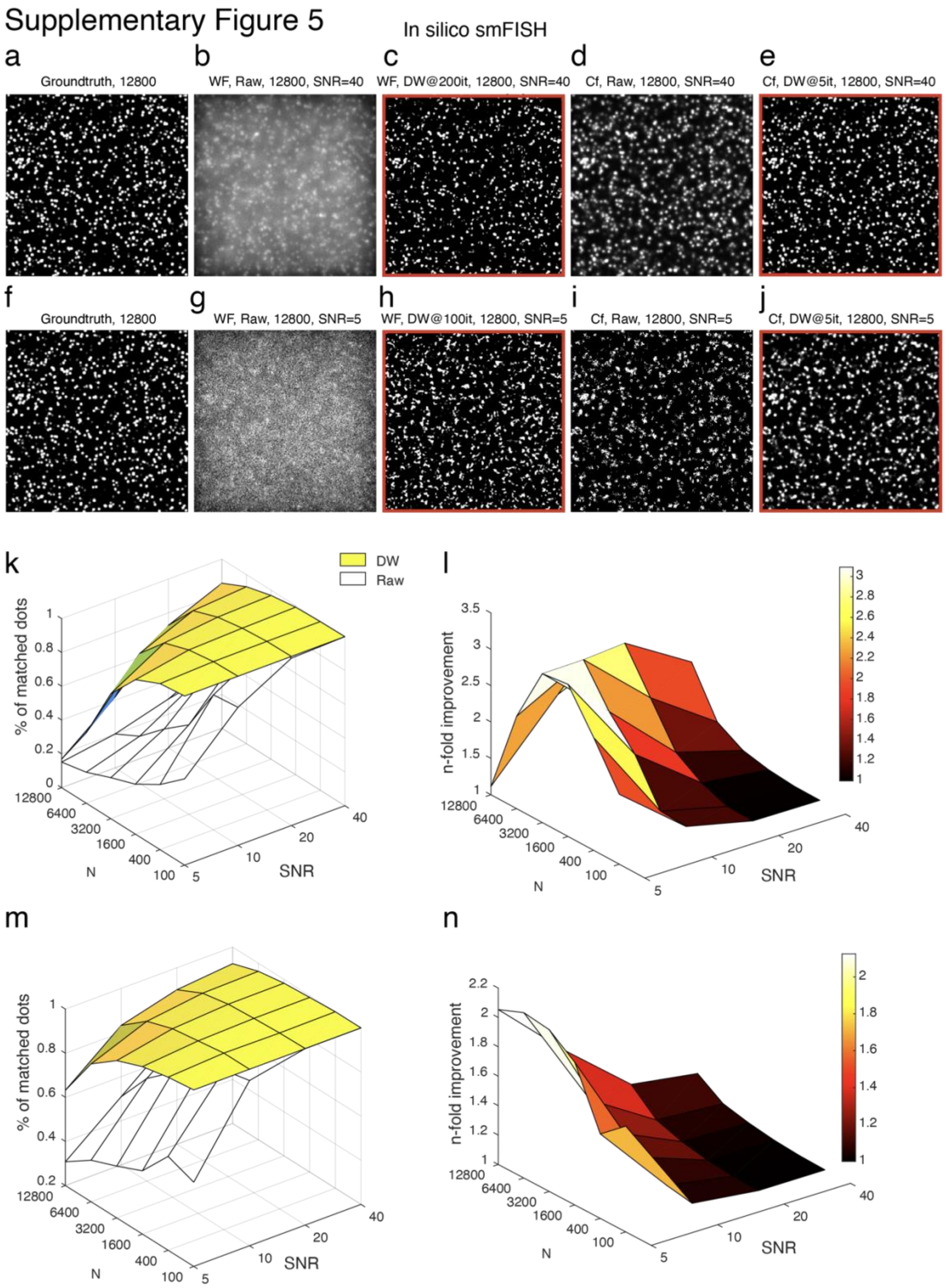


**Supplementary Fig. 5.** Deconwolf (DW) is superior to confocal imaging for the detection of diffraction-limited dots in highly crowded images. (**a**) Single slice of a z-stack image containing a high density of in silico generated diffraction-limited dots. (**b**) As in (a) but

applying noise (signal-to-noise ratio, SNR=40) to simulate an image that would be obtained by applying smFISH to visualize the transcripts of a highly expressed gene. (**c**) Same image as in (b) after deconvolution with DW (200 iterations, it). (**d**) As in (b) but simulating a confocal (Cf) image with an SNR of 40. (**e**) Same image as in (d) after deconvolution with DW (5 iterations, it). (**f-j**) Same series as in (a-e) but using an SNR of 5. (**k**) Dot detection efficiency in widefield images with (DW) or without (Raw) deconvolution with DW at varying number of dots per image and SNR values. The dot detection efficiency was calculated as the percentage of the overlap between the local maxima identified in the raw or deconvolved images and the local maxima of the dots in the corresponding ground truth images exemplified in (a) and (f). (**l**) Extent of improvement (calculated as fold change) in dot detection efficiency comparing the DW and Raw landscapes in (k). (**m, n**) As in (k, l) but for confocal images exemplified in (d, e) and (i, j). DW-deconvolved images are framed in red. All micrographs are from a single experiment.

Supplementary Figure 6

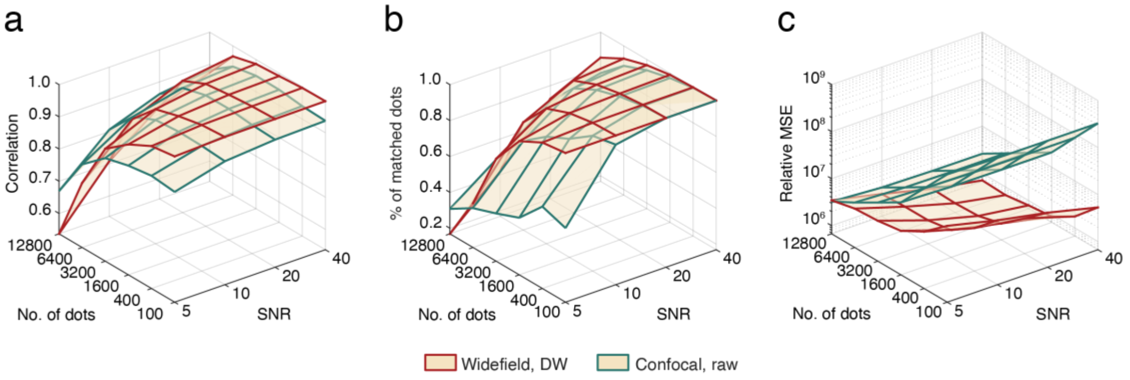


**Supplementary Fig. 6.** Deconwolf (DW) is superior to confocal imaging for the detection of diffraction limited dots in highly crowded images. (**a**) Correlation between the pixel values in the ground truth z-stack images of high-density diffraction limited dots (as exemplified in **Supplementary Fig. 5a**) and the corresponding simulated widefield images deconvolved with DW (as exemplified in **Supplementary Fig. 5c and 5h**) or by confocal microscopy (as exemplified in **Supplementary Fig. 5d and 5i**) at varying number of dots per image and signal-to-noise (SNR) values. (**b**) Dot detection efficiency in widefield DW-deconvolved images or in confocal images at varying number of dots per image and SNR values. The dot detection efficiency was calculated as the percentage of the overlap between the local maxima identified in the raw or deconvolved images and the local maxima of the dots in the corresponding ground truth images. (**c**) Relative mean squared error (MSE) for widefield DW-deconvolved images (as exemplified in **Supplementary Fig. 5c and 5h**) or confocal images (as exemplified in **Supplementary Fig. 5d and 5i**) at varying number of dots per image and SNR values. The images are scaled to have a mean value of 1,000 before calculating the mean squared error.

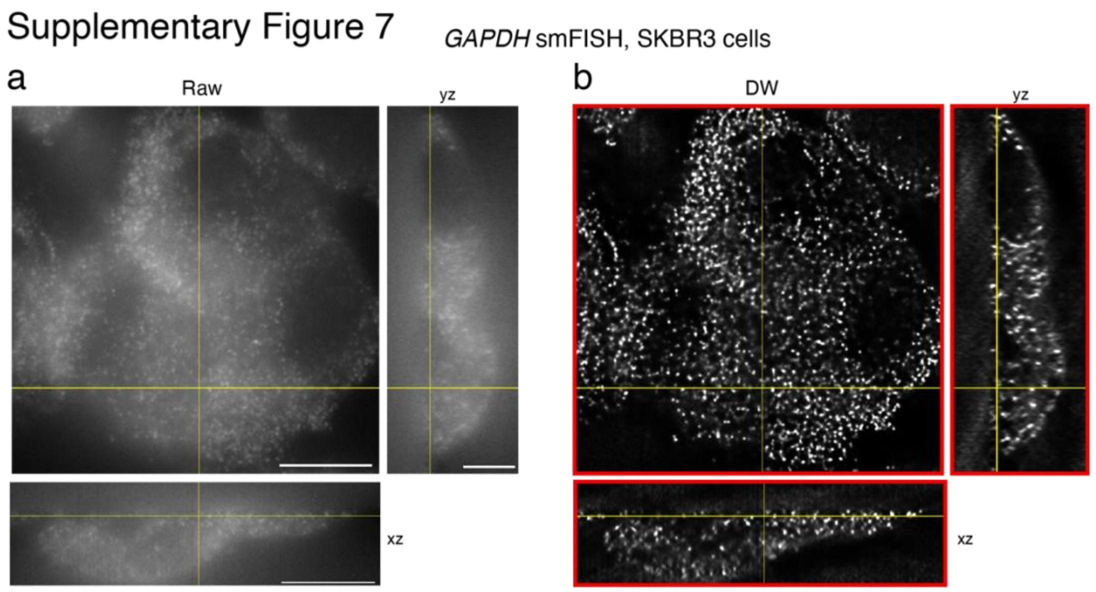


**Supplementary Fig. 7.** Deconwolf (DW) drastically improves the visualization of individual transcripts in crowded smFISH images. (**a**) xy view (large square) as well as orthogonal views (xz and yz) of a single focal plane of a z-stack image from SKBR3 human breast carcinoma cells subjected to smFISH with a probe for *GAPDH* gene transcripts and imaged on a widefield microscope using a 100x oil objective. (**b**) As in (a) but after deconvolution with DW. DW-deconvolved images are framed in red. All micrographs are from a single experiment.

Supplementary Figure 8

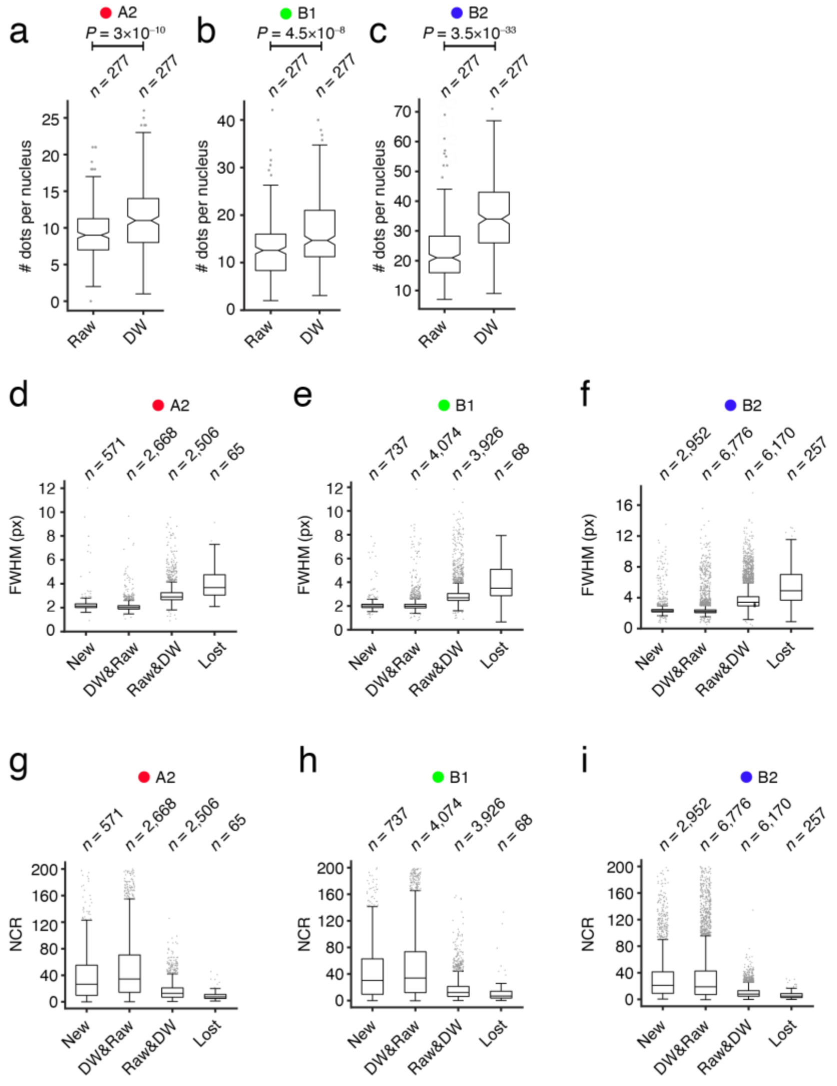

**Supplementary Fig. 8.** Deconwolf (DW) improves the sensitivity and specificity of dot detection in iFISH images. (**a-c**) Distributions of the total number of iFISH dots per nucleus for DNA loci located in the indicated A/B subcompartment, either in non-deconvolved (Raw) images or in DW-deconvolved images from the same dataset from which the images shown in (b) were created (see **Fig. 4j, k**). *n*, number of cells analyzed. *P*, Wilcoxon test, two-tailed (**d-f**) Distributions of the full width at half maximum (FWHM) values of the dots analyzed in (c-e). New: dots detected only in DW-deconvolved images. Lost: dots detected in raw images, but not after applying DW. DW&Raw: dots detected after applying DW, that were also present in the corresponding raw images. Raw&DW: dots detected in the raw images that were also detected in the corresponding images after deconvolution with DW. *n*, number of dots analyzed. (**g-i**) As in (f-h) but for nuclear-contrast-ratio (NCR) values of the iFISH dots. In all the

boxplots in (c-k), each boxplot extends from the 25th to the 75th percentile, the horizontal bar represents the median, and whiskers extend from $-1.5\times$IQR to $+1.5\times$IQR from the closest quartile, where IQR is the inter-quartile range. Grey dots, outliers.

Supplementary Figure 9

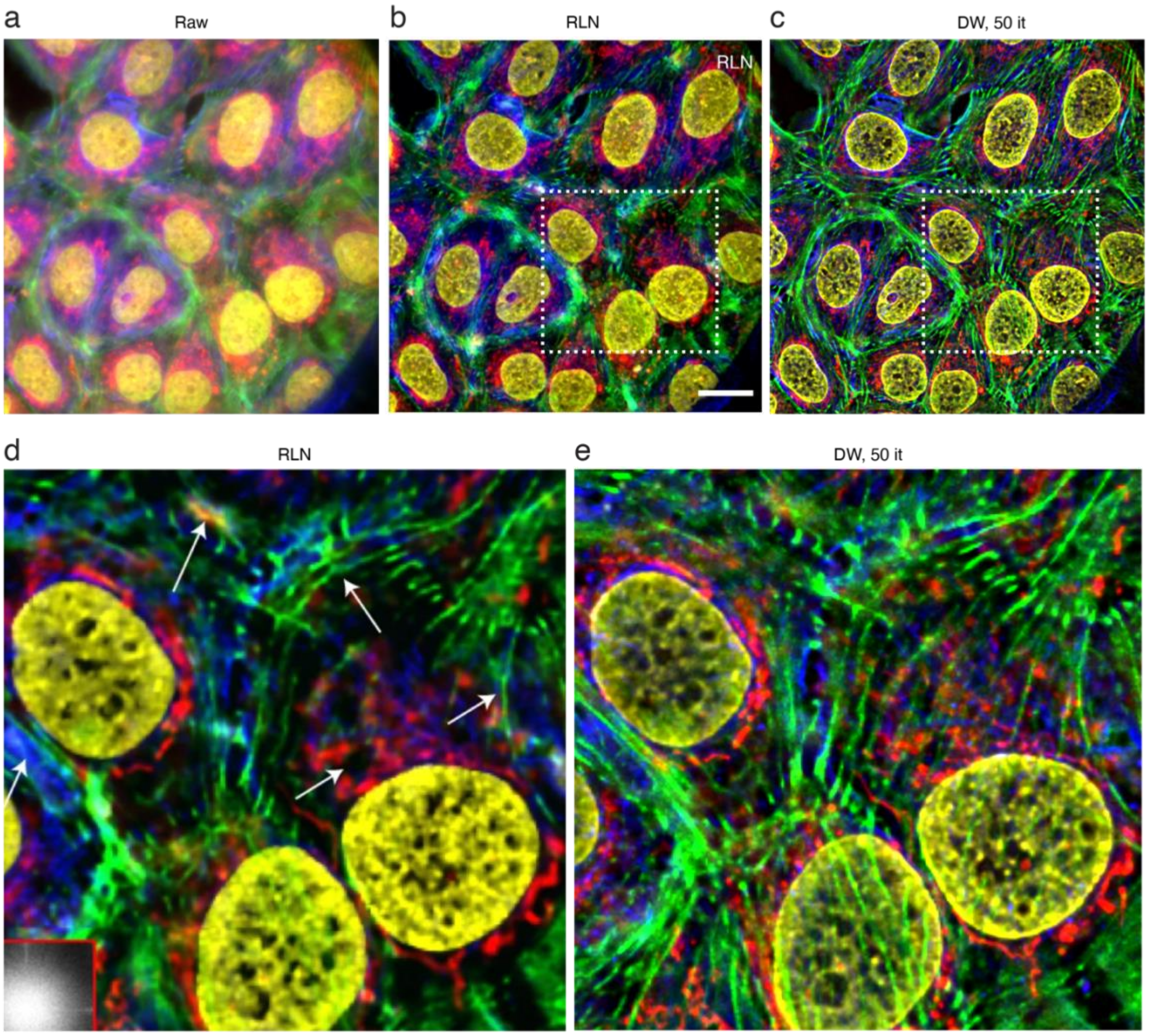


**Supplementary Fig. 9.** Comparison between the Richardson-Lucy Network (RLN) method[4] and Deconwolf (DW). (**a**) Widefield image of U-2 OS cells stained for mitochondria (red), actin (green) and tubulin (blue). We downloaded the image from the original paper describing the RLN method[4] (see Fig. 5 in the RLN paper) and processed it in ImageJ (v1.54f) for visualization purposes. (**b**) As in (a) but after RNL processing. We made a screenshot of the image shown in Fig. 5 of the published paper since only raw data are provided in the paper. (**c**) As in (a) but after deconvolution with DW (50 iterations, it). (**d, e**) Zoom-in views of the corresponding regions in (b) and (c) encircled by the dashed white squares.

# 2. Supplementary Notes

### 1. Brief introduction to fluorescence microscopy image deconvolution

Image deconvolution can be described as an inverse problem, where $y = f(x)$ is known and the aim is to find out which $x$ generated $y$. In fluorescence microscopy, $x$ represents the emission pattern from the specimen visualized under the microscope, whereas $y$ represents the image that is acquired by the detector, including noise. The whole image formation process is represented by $f$, *i.e.*, how the emission pattern is converted to an image throughout the microscope[3]. Due to nonlinearities of typical imaging systems, such as optical aberrations and varying refractive index of the samples, $f$ can only be approximated. Richardson[5] and, independently, Lucy[6] devised an iterative expectation-maximization (EM) procedure to solve the image deconvolution problem under the assumption that $y = h \times x + n$, where $\times$ denotes a linear convolution operator and $n$ is Poissonian noise (from now on, we refer to this method as the Richardson-Lucy or RL method). The RL method requires that the kernel (or point spread function, PSF), $h$, is known, but in practice $h$ is only approximated. In RL, the likelihood always increases from one iteration to the next one[6]. However, the presence of noise can cause the RL method to diverge significantly from the true solution if it is iterated excessively[6]. A known method to tame this is to pre-filter the observed image with a Gaussian kernel[7]. Total variation minimization (TV) as well as Tikhonov regularization are also known techniques that can be used to reduce the effect of noise on deconvolution methods[8].

### 2. Resolution

For an image-forming system, resolution is typically defined as the smallest distance, $d$, separating two points before they blend. In widefield microscopy, the Rayleigh criterion $d = 0.61\lambda NA$ (where $\lambda$ is the light wavelength and $NA$ is the objective numerical aperture), defined as the distance from the maxima of Airy disk to the first minima, is often used for this purpose. When two imaged points are separated by the Rayleigh distance there will be an intensity drop in between them, making the two points still separable. The Sparrow criterion suggests an even smaller value, specified by $d = 0.5\lambda NA$, as the distance where there is no intensity drop between the two points[9].

Several demonstrations have shown that deconvolution can increase the resolution of microscopy images[10], although the improved resolution depends on the noise level and how well the system is characterized. The resolution of deconvolved images cannot be assessed in

the same way as for widefield images. For example, the sharpness of edges can be used to estimate the resolution of widefield images, but the same cannot be done on deconvolved images since they often have very sharp edges which would misleadingly indicate high resolution. Importantly, deconvolution cannot increase the resolution of extended regions with smooth contrast but, for the same image, can shrink point-like signals to single pixels. Since DW is based on the RL method (see **Supplementary Note 1**), we expected it to share the same resolution improvement limits as other deconvolution software based on the same principles. To show that this is the case, we generated a point spread function (PSF) with $\lambda = 500$, $NA = 1.45$, and $ni = 1.515$. For such a system, the Sparrow resolution is $r = 0.5\lambda NA \cong 172.4\ nm$. We then simulated an image with two points separated by 172 nm and a pixel size of 86 nm, i.e., just below the Sparrow resolution. This produced an image with a single intensity maximum just between the two points as seen in **Supplementary Notes Fig. 1** below (left). After deconvolution, the two points become separated (left), demonstrating that, indeed, deconvolution can improve the resolution beyond the Sparrow limit. More in-depth descriptions on how deconvolution can increase resolution can be found in refs. [10,11].

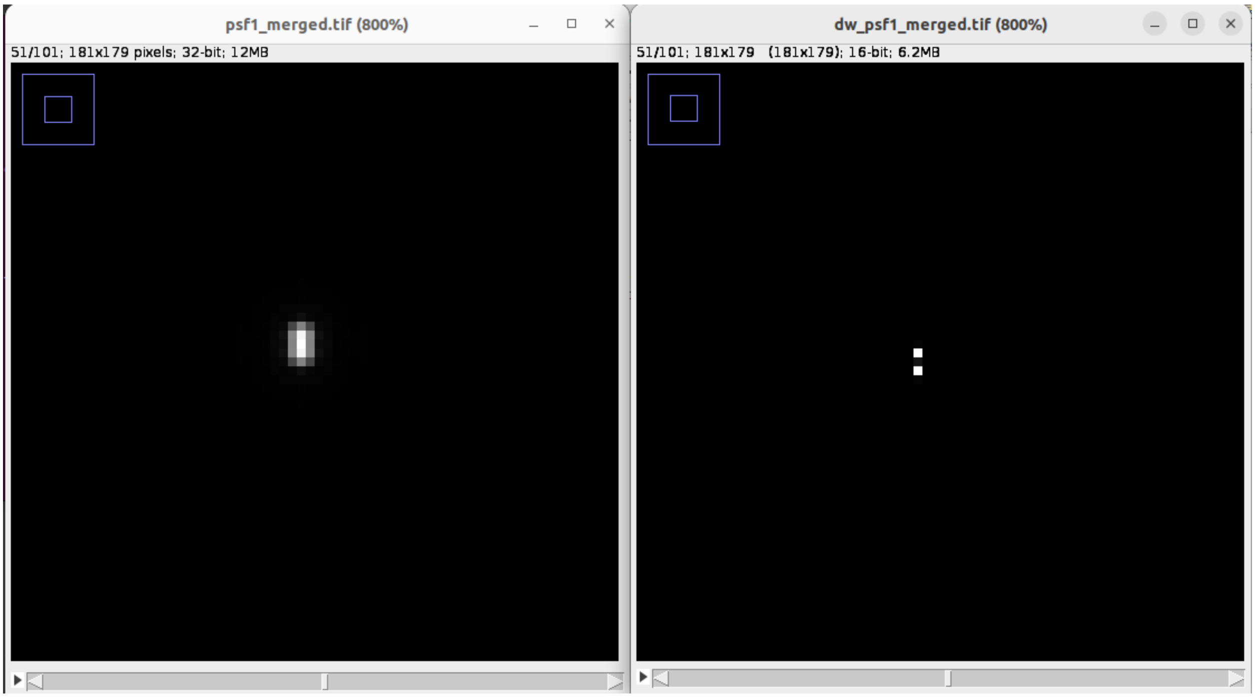


**Supplementary Notes Fig. 1.** Left: Simulation of two points separated by less than the Sparrow resolution, i.e., the intensity between the two points is higher than at each point. Right: same image on the left after deconvolution by DW.

**3. PSF calculation**

A point spread function (PSF) describes how a single bright point, or point source, will appear in a focus stack of a microscopy image[12]. For a widefield microscope, the ideal PSF has an hourglass shape along the axial direction and is symmetric around the lateral plane. The purpose of deconvolution is to reverse the additive PSF patterns for all light sources in a sample and convert them back to single bright points, at least to some extent. It should be noted that perfect deconvolution does not exist: even if it were possible to record noise-free images using theoretically perfect objectives, information would be lost during imaging (which can be seen as a loss of frequencies in the Fourier domain) and could not be retrieved again[3].

Like any other deconvolution tool with a practical run time, DW uses a PSF model that is constant over the image and does not change over the image field or along the axial direction. A typical relaxation of that criteria is to use PSF that varies with focus depth[13]. Such approach can theoretically produce better deconvolution results but will have a much higher computational cost and will require an extensive quantification of the imaging system. For most of the images displayed in this paper, we used PSFs calculated using the so-called Born and Wolf model (BW)[3,14]. Arguably, this is a convenient choice since it relies on only few key parameters, which are readily available for any microscope system. However, the model is derived under several assumptions, including perfect optics (which gives radial symmetry and symmetry around the lateral plane) and low numerical aperture (NA). In practice this works well for relatively simple images, such as smFISH images in which the signal is represented by (near-)diffraction limited dots. Better deconvolution results might however be obtained when the PSF can be fine-tuned to a specific microscope and objective and to a specific sample. A notable generalization of the BW model can be found in the Gibson and Lanni model (GL)[15], which incorporates mismatches between actual and ideal imaging conditions, such as deviations of refractive index between the immersion media and the sample. Although the GL model has shown good agreement with experimental data, it requires further parameters that are typically not easy to set. Alternatively, the PSF can be measured from experimental data[12,14,16]. This can be done by imaging one or more fluorescent beads (representing point sources) and then averaging the images of those, possibly also considering the size of the beads. Ideally the beads should be placed in real biological samples, since the samples themself contribute to the optical environment by having varying refractive index. In our experience, a simple theoretical PSF is often good enough for smFISH and high-resolution DNA FISH images, but for other microscopes and samples the reverse might hold true.

While the focus in this work has been on widefield images, we have also implemented and used PSFs for confocal and STED super-resolution microscopy. The PSF from a confocal

microscope is more challenging to describe than the PSF of widefield microscopes since it combines two PSFs: one describing the illumination and the other describing the excitation, as well as an aperture opening of variable size. For STED, we have used a Lorentzian in the lateral plane combined with a Gaussian in the axial direction. Details about the generation of PSFs for confocal and STED are described later in this Supplementary Note.

To generate a PSF from the BW model involves solving the following integral for each radii, $r$, and z-value (symmetric also around $z = 0$):

$$h(r,z) = \left| \int_0^1 \rho J_0(nkr\rho) \exp(-ik\rho^2 zn^2/2)\, d\rho \right|^2 \tag{1}$$

where $J_0$ is the first Bessel function of the first kind, $k = 2\pi/\lambda$ and $n = NA/ni$.
To calculate the PSF, it is common to first sample this integral over a 2D grid to create a map, $M(r,z)$ at discrete points and then sample this grid for each pixel coordinate $(x,y,z)$ of the PSF. This approach is used in the PSF Generator (PG) and is described in other implementations as well[13,14]. A consequence of the sampling approach described above is that the value of the PSF for the central pixel will be:

$$PSF(0,0,0) = h(0,0) \tag{2}$$

which is always an over estimation of the true value:

$$PSF(0,0,0) = \int_{-dx/2}^{dx/2} \int_{dy/2}^{dy/2} h(r(x,y),0) dxdy \tag{3}$$

since $h(r,z)$ attains its maximum value at (0,0). In particular, the larger the pixel size, $dx$, is in relation to the Airy Disk diameter, $AU$, the worse the error will be, i.e., the error grows with $dx/AU$. As a result, the PSF will be given an exaggerated value at the central pixel, causing an unwanted regularization.

The PSF Calculator (PC) used in DW first calculates the BW integral over an oversampled grid of $(r,z)$ values to produce $M(r,z)$. In a second step it integrates this map over each pixel. To further increase the precision and reduce biases a Lanczos-5 interpolation

with symmetry around $r = 0$ ($L_5$) is used. At a given $z$ this means that the pixel value can be calculated as:

$$PSF(x,y) = \int_{x-0.5dx}^{x+0.5dx} \int_{y-0.5dy}^{y+0.5dy} L_5\big(M, r(x,y)\big) dy dx \tag{4}$$

We found that a 61-point Gauss-Kronrod rule worked very well for the BW integral while a 15-point Gauss-Kronrod rule was faster when integrating over pixels. For numerical integration, DW uses the GNU Scientific Library[17], while the code is parallelized using POSIX Threads.

The confocal PSF can be described as a combination of the excitation PSF, $P_{exc}$, and the emission PSF, $P_{emi}$, together with a pinhole, $Ph$, with a size often expressed in Airy Units (AU)[18]. We start with the PSF model (for details, see ref. [19]):

$$P(x,y,z;\lambda) = \int\int U\big(k_x,k_y\big) \exp[ik_z(kx,ky)z] \exp\big[i(k_x x + k_y y)\big] d_{k_x} d_{k_y} \tag{5}$$

where $U$ is the pupil function which depends on $NA$, $ni$ and $\lambda$. To restrict the $P_{exc}$ by the aperture opening we calculate:

$$\hat{P}_{exc} = P_{exc} * Ph \tag{6}$$

where $*$ denotes convolution in the lateral plane. Finally, the PSF can be expressed as:

$$PSF = \hat{P}_{exc} P_{emi} \tag{7}$$

The convolutions are evaluated in the Fourier domain using a padded and possibly over sampled representation.

In STED microscopy, the PSF is typically describes as a Lorentzian in the lateral plane[18]:

$$L(x,y|\gamma) = \frac{1}{2\pi} \frac{\gamma}{((x-x_0)^2 + (y-y_0)^2 + \gamma^2)^{3/2}} \tag{8}$$

depending on a single parameter, $\gamma$. For DW, we have extended the PSF to 3D by combining it with a Gaussian, to produce the following model:

$$PSF(x,y,z|\gamma,\sigma) = L(x,y|\gamma)G(z|\sigma) \quad (9)$$

The parameters $\gamma$ and $\sigma$ can be set by matching the axial FWHM, $fwhm_L$, and the lateral FWHM, $fwhm_L$, of isolated dots to the model, i.e.:

$$\sigma = \frac{fwhm_A}{2\sqrt{2\log(2)}} \quad (10)$$

$$\gamma = \frac{fwhm_L}{2\sqrt{2^{2/3} - 1}} \quad (11)$$

**4. Handling image boundaries**

In widefield microscopy, a single field of view (FOV) is limited in extent and its content will, in most cases, also consist of out-of-focus light from the surroundings. A naive implementation of the RL method in the Fourier domain, without considering the boundaries, implicitly assumes that the image wraps around all dimensions causing bleed-through from top to bottom, left to right, etc., without considering that some of the detected light might have a source outside of the image boundaries. Many test images appear to be generated in this way, and in that case this information needs to be communicated to DW. For example, the synthetic microtubule and hollow bar images shown in **Extended Data Fig. 1e, f** were deconvolved assuming that the image boundaries wrap. For real data, the naive approach can give rise to severe deconvolution artifacts and several approaches have been proposed to alleviate this problem. Two commonly used approaches are 1) padding, assuming that the image is constant outside of what is imaged; and 2) apodization, where the image pixels slowly fade towards the edges[20]. Unfortunately, apodization comes at the cost of losing information close to the edges of the image, while padding poses the risk of introducing more artifacts if the padding value is too different from the actual field outside of the FOV. More elegant solutions exist in other frameworks (see https://hal.science/hal-00691249) but are not compatible with Poissonian noise. DW uses the Bertero-Boccacci method (BBM)[21] originally described for astronomic 2D images and RL deconvolution. Computationally it starts with zero-padding of the image and the addition of an extra weight matrix, but unlike zero-padding it allows some of the light to be explained by sources outside of the FOV. DW uses BBM and extends it to 3D

deconvolution. Importantly, the problem size does not need to be doubled in each dimension (as also pointed out in the original BBM paper[21]) when the PSF is smaller than the image. For an image of size $M \times N \times P$ and a PSF of size $Q \times R \times S$, a work of size equal to $(M + Q) \times N + R \times (P + S)$ is enough (typically, this is only 50% more pixels than the original image). While we initially implemented BBM for the RL method, we have also found that there is nothing that hinders it from being used with accelerated methods (AVE, EVE or SHB). Unless specified, DW uses the extended BBM method as default to handle image boundaries.

## 3. Supplementary References


1. Danie Sage. PSF Generator - Icy – Open Source Image Processing Software. http://icy.bioimageanalysis.org/plugin/psf-generator/.
2. Nguyen, H. Q. *et al.* 3D mapping and accelerated super-resolution imaging of the human genome using in situ sequencing. *Nat. Methods* **17**, 822–832 (2020).
3. Born, M., Wolf, E. & Bhatia, A. B. *Principles of Optics: Electromagnetic Theory of Propagation, Interference, and Diffraction of Light*. (Cambridge University Press, Cambridge, 2019).
4. Li, Y. *et al.* Incorporating the image formation process into deep learning improves network performance. *Nat. Methods* **19**, 1427–1437 (2022).
5. Richardson, W. H. Bayesian-Based Iterative Method of Image Restoration*. *J. Opt. Soc. Am.* **62**, 55–59 (1972).
6. Lucy, L. B. An iterative technique for the rectification of observed distributions. *Astron. J.* **79**, 745 (1974).
7. Verveer, P. J., Gemkow, M. J. & Jovin, T. M. A comparison of image restoration approaches applied to three-dimensional confocal and wide-field fluorescence microscopy. *J. Microsc.* **193**, 50–61 (1999).
8. Zunino, A., Benvenuto, F., Armadillo, E., Bertero, M. & Bozzo, E. Iterative deconvolution and semiblind deconvolution methods in magnetic archaeological prospectingDeconvolution in magnetic prospecting. *Geophysics* **74**, L43–L51 (2009).
9. Yen, A. Rayleigh or Abbe? Origin and naming of the resolution formula of microlithography. *J. MicroNanolithography MEMS MOEMS* **19**, (2020).
10. Zhao, W. *et al.* Sparse deconvolution improves the resolution of live-cell super-resolution fluorescence microscopy. *Nat. Biotechnol.* **40**, 606–617 (2022).

11. Ahi, K. & Anwar, M. Developing terahertz imaging equation and enhancement of the resolution of terahertz images using deconvolution. in (eds. Anwar, M. F., Crowe, T. W. & Manzur, T.) 98560N (Baltimore, Maryland, United States, 2016). doi:10.1117/12.2228680.
12. McNally, J. G., Karpova, T., Cooper, J. & Conchello, J. A. Three-dimensional imaging by deconvolution microscopy. *Methods San Diego Calif* **19**, 373–385 (1999).
13. Li, J., Xue, F., Qu, F., Ho, Y.-P. & Blu, T. On-the-fly estimation of a microscopy point spread function. *Opt. Express* **26**, 26120 (2018).
14. Kirshner, H., Aguet, F., Sage, D. & Unser, M. 3-D PSF fitting for fluorescence microscopy: implementation and localization application. *J. Microsc.* **249**, 13–25 (2013).
15. Gibson, S. F. & Lanni, F. Experimental test of an analytical model of aberration in an oil-immersion objective lens used in three-dimensional light microscopy. *J. Opt. Soc. Am. A* **9**, 154–166 (1992).
16. Hiraoka, Y., Sedat, J. W. & Agard, D. A. Determination of three-dimensional imaging properties of a light microscope system. Partial confocal behavior in epifluorescence microscopy. *Biophys. J.* **57**, 325–333 (1990).
17. *GNU Scientific Library Reference Manual: For GSL Version 1.12*. (Network Theory, Bristol, 2009).
18. Alvelid, J. & Testa, I. Stable stimulated emission depletion imaging of extended sample regions. *J. Phys. Appl. Phys.* **53**, 024001 (2020).
19. Hanser, B. M., Gustafsson, M. G. L., Agard, D. A. & Sedat, J. W. Phase retrieval for high-numerical-aperture optical systems. *Opt. Lett.* **28**, 801 (2003).
20. Sage, D. *et al.* DeconvolutionLab2: An open-source software for deconvolution microscopy. *Methods San Diego Calif* **115**, 28–41 (2017).

21. Bertero, M. & Boccacci, P. A simple method for the reduction of boundary effects in the Richardson-Lucy approach to image deconvolution. *Astron. Astrophys.* **437**, 369–374 (2005).
