# Supplementary material for: Deconwolf enables high-performance deconvolution of widefield fluorescence microscopy images
Source: Nat Methods. 2024 Jun 6;21(7):1245–56. doi: 10.1038/s41592-024-02294-7 (PMC11239506; doi:10.1038/s41592-024-02294-7)
Supplement: Supplementary file 2 — Reporting Summary [file 41592_2024_2294_MOESM2_ESM.pdf]

nature research

Corresponding author(s): Magda Bienko

Last updated by author(s): Mar 25, 2024

Reporting Summary

Nature Research wishes to improve the reproducibility of the work that we publish. This form provides structure for consistency and transparency in reporting. For further information on Nature Research policies, see our Editorial Policies and the Editorial Policy Checklist.

Statistics

For all statistical analyses, confirm that the following items are present in the figure legend, table legend, main text, or Methods section.

n/a	Confirmed	
☐	☒	The exact sample size (*n*) for each experimental group/condition, given as a discrete number and unit of measurement
☐	☒	A statement on whether measurements were taken from distinct samples or whether the same sample was measured repeatedly
☐	☒	The statistical test(s) used AND whether they are one- or two-sided<br>*Only common tests should be described solely by name; describe more complex techniques in the Methods section.*
☒	☐	A description of all covariates tested
☒	☐	A description of any assumptions or corrections, such as tests of normality and adjustment for multiple comparisons
☐	☒	A full description of the statistical parameters including central tendency (e.g. means) or other basic estimates (e.g. regression coefficient) AND variation (e.g. standard deviation) or associated estimates of uncertainty (e.g. confidence intervals)
☐	☒	For null hypothesis testing, the test statistic (e.g. *F*, *t*, *r*) with confidence intervals, effect sizes, degrees of freedom and *P* value noted<br>*Give P values as exact values whenever suitable.*
☒	☐	For Bayesian analysis, information on the choice of priors and Markov chain Monte Carlo settings
☒	☐	For hierarchical and complex designs, identification of the appropriate level for tests and full reporting of outcomes
☒	☐	Estimates of effect sizes (e.g. Cohen's *d*, Pearson's *r*), indicating how they were calculated

Our web collection on statistics for biologists contains articles on many of the points above.

Software and code

Policy information about availability of computer code

Data collection	For image acquisition: 1) NIS Elements software (Nikon) to operate the Ti-E microscope (Nikon) that we used to collect widefield microscopy images; 2) Leica Application Suite X (LAS X 3.5.7.32225 version) for confocal imaging; 3) ZEN v2.3 (Zeiss) microscopy software to acquire ISST data.
Data analysis	--For smFISH and iFISH dot detection: DOTTER (v0.598, https://github.com/elgw/dotter)<br>--For smFISH data analysis: https://github.com/ggirelli/deconwolf-tissue-smFISH, nuclei segmentation: https://github.com/elgw/pixelClassifier<br>--For ISST data analysis: https://github.com/Moldia/iss_starfish/, http://github.com/spacetx/starfish<br>--For deconvolution: 1) DeconvolutionLab2 (v2.1.2); 2) Deconwolf 0.0.20 as well as more recent versions; 3) Huygens (v17.04); 4) Nikon NIS Elements AR (v5.02.0); RedLionfish (v0.9)<br>--For visual rendering of the image shown in Fig 5 of the RLN paper (https://doi.org/10.1038/s41592-022-01652-7): ImageJ (v1.54f)<br>--The entire DW package can be freely downloaded from https://deconwolf.fht.org/ and is also available at https://github.com/elgw/deconwolf/.

For manuscripts utilizing custom algorithms or software that are central to the research but not yet described in published literature, software must be made available to editors and reviewers. We strongly encourage code deposition in a community repository (e.g. GitHub). See the Nature Research guidelines for submitting code & software for further information.

Data

Policy information about availability of data

All manuscripts must include a data availability statement. This statement should provide the following information, where applicable:

- Accession codes, unique identifiers, or web links for publicly available datasets
- A list of figures that have associated raw data
- A description of any restrictions on data availability

--The images of synthetic microtubules, fluorescent rods, and C. elegans whole embryo used for DW benchmarking can be downloaded from http://bigwww.epfl.ch/deconvolution/index.html - data.
--Source data to reproduce all the figures, including raw and deconvolved images, imaging and deconvolution settings, and tabulated data to reproduce all the plots are available on Figshare at: https://figshare.com/s/64d00b42a5d0c5178c19.
--Tabulated data to reproduce smaller plots in the main Figures and Extended Data Figures are also available as a separate Source Data file.
--The RLN image shown in Supplementary Fig. 9a was downloaded from https://doi.org/10.1038/s41592-022-01652-7 7 (see Fig. 5 in the paper).
--iFISH probes were designed using the human genome assembly GRCh38/hg38.
--smFISH probes were designed using the ENSEMBL transcript IDs listed in Supplementary Table 1.

Field-specific reporting

Please select the one below that is the best fit for your research. If you are not sure, read the appropriate sections before making your selection.

☒ Life sciences ☐ Behavioural & social sciences ☐ Ecological, evolutionary & environmental sciences

For a reference copy of the document with all sections, see nature.com/documents/nr-reporting-summary-flat.pdf

Life sciences study design

All studies must disclose on these points even when the disclosure is negative.

Sample size	All images analyzed were obtained through single experiments (i.e., no experimental replicate was performed) since the purpose of the study was to test and validate Deconwolf on different types of images acquired through different microscopy settings. Therefore, no sample size calculation was performed a priori.
Data exclusions	In all smFISH data analysed, unspecific fluorescence dots with FWHM < 200 nm or > 800 nm were excluded since smFISH generates near-diffraction limited dots with FWHM distribution between 200 and 800 nm.
Replication	--No experimental replicates were performed, i.e., all micrographs shown and corresponding analyses come from a single biological experiment.<br>--For deconvolution, the exact command line to replicate the deconvolution is included in the log files uploaded with the original images and the deconvolved images to Figshare: https://figshare.com/s/64d00b42a5d0c5178c19.
Randomization	No randomizations were performed. No covariate analysis for variables such as sample sex, age, type was performed since we did not analyze multiple image datasets coming from multiple samples of the same type but instead compared different deconvolution methods on the same image obtained from a single sample (cells or tissue section) in a single experiment.
Blinding	No blinding was used and is arguably not needed since we used automated scripts for reported quantities.

Reporting for specific materials, systems and methods

We require information from authors about some types of materials, experimental systems and methods used in many studies. Here, indicate whether each material, system or method listed is relevant to your study. If you are not sure if a list item applies to your research, read the appropriate section before selecting a response.

Materials & experimental systems

n/a	Involved in the study	
☐	☒	Antibodies
☐	☒	Eukaryotic cell lines
☒	☐	Palaeontology and archaeology
☒	☐	Animals and other organisms
☒	☐	Human research participants
☒	☐	Clinical data
☒	☐	Dual use research of concern

Methods

n/a	Involved in the study	
☒	☐	ChIP-seq
☒	☐	Flow cytometry
☒	☐	MRI-based neuroimaging

Antibodies

Antibodies used

--Anti-GFAP (Atlas Antibodies, cat. no. AMAb91033), 1:500 dilution (vol/vol)
--Anti-Nup153 (Abcam cat. no. ab24700), diluted to 1 µg/mL
--Anti-tubulin (Sigma-Aldrich, cat. no. T6074), 1:250 dilution (vol/vol)
--Goat anti-mouse secondary antibody coupled with AlexaFluor 555 (Thermo Fisher Scientific, cat. no. A-21424), 1:800 dilution (vol/vol)
--Goat anti-mouse secondary antibody coupled with AlexaFluor 555 (Abcam, cat. no. ab150118), 1:400 dilution (vol/vol)
--Goat anti-mouse IgG secondary antibody (Merck, cat. no. ST635P-1002-500UG), 1:400 dilution) (vol/vol).

Validation

Anti-GFAP antibodies from Atlas Antibodies (cat. no. AMAb91033) were validated by the manufacturer by comparison to RNA-seq data from high- and low-expressing human tissues.

Anti-Nup153 antibodies (Abcam, cat. no. ab24700) are recommended for ICC/IF on human cells/tissues and can also recognize other NPC polypeptides (p250 and p62) apart from Nup153, as stated on Abcam website (https://www.abcam.com/products/primary-antibodies/nup153-antibody-qe5-ab24700.html).

Anti-tubulin antibodies (Sigma-Aldrich, T6074) are recommended for ICC/IF and Western blot and have undergone Enhanced Validation by Independent Antibodies as described on the Sigma-Aldrich website: https://www.sigmaaldrich.com/IT/it/technical-documents/technical-article/protein-biology/immunohistochemistry/antibody-enhanced-validation

Eukaryotic cell lines

Policy information about cell lines

Cell line source(s)

We purchased SKBR3 cells from ATCC (cat. no. HTB-30), hTERT RPE-1 cells from ATCC (cat. no CRL-4000), U-2 OS cells from CLS Cell Lines Service (cat. no. 300174), and HAP1 cells from Horizon Discovery (cat. no. C859).

Authentication

We did not authenticate any of the cell lines used.

Mycoplasma contamination

The cell lines used were tested for Mycoplasma contamination and were negative.

Commonly misidentified lines
(See ICLAC register)

None of the cell lines used is included in the ICLAC database of commonly misidentified cell lines.
